# Clonal articular cartilage progenitors outperform mesenchymal stromal cells in mitigating post-traumatic osteoarthritis

**DOI:** 10.1016/j.ocarto.2026.100878

**Published:** 2026-09-25

**Authors:** Heidi Kloser, Parvathy Thampi, Lynn Pezzanite, Taryn Boxleitner, Lyndah Chow, Steve Dow, Katie J. Sikes, Kelly Santangelo, Laurie Goodrich, Brian Johnstone

**Affiliations:** a Colorado State University, Fort Collins, CO, USA; b Oregon Health & Science University, Portland, OR, USA

**Keywords:** Destabilization of the medial meniscus (DMM), Articular chondroprogenitors, Mesenchymal stromal cells, Behavior/mobility, Stromal cell therapies, Post-traumatic osteoarthritis

## Abstract

**Objective:**

This study aimed to compare the functional, pathological, and immunomodulatory properties of articular chondroprogenitors (ACPs) with those of bone-marrow-derived mesenchymal stromal cells (MSCs) in preventing the progression of post-traumatic osteoarthritis (PTOA) after destabilization of the medial meniscus (DMM) injury in a mouse model.

**Method:**

ACP and MSC were isolated from three horses and assessed for their immunomodulatory capacity in vitro. Aliquots of selected ACP clones and MSCs were injected intra-articularly into male mice 1-week post destabilization of the medial meniscus (DMM). The functional mobility of the mice before the injury and at weeks 2, 4, and 8 post-injury was assessed by two methods: ANY-maze™ voluntary mobility monitoring and DigiGait™ compulsory gait analysis. Upon euthanasia, joints were collected for histological scoring.

**Results:**

In vitro assessments demonstrated that ACPs and MSCs had anti-inflammatory properties: both treatments produced lower levels of pro-inflammatory cytokines, while only the ACPs produced more anti-inflammatory cytokines than controls. In vivo, both ACPs and MSCs lessened the effects of DMM on mobility. Histological scoring revealed significant reductions of PTOA in the medial compartment with ACPs relative to both MSCs and vehicle controls. Overall, ACPs effectively reduced PTOA progression to a greater extent than MSCs.

**Conclusion:**

Our findings demonstrated that both equine ACP and MSC treatments provided symptom modification over vehicle controls in a short-term animal study. Notably, ACPs may be more efficacious than MSCs at reducing structural joint pathology.

## 1 Introduction

Post-traumatic osteoarthritis (PTOA) is a debilitating joint disease for which there is still a need for effective interventions to halt or reduce joint degeneration after injury. PTOA cases account for approximately 12% of all OA cases [1,2]. Individuals who have experienced a major joint injury (e.g. ligament and cartilage injury) face a 25% risk of developing PTOA, with this risk increasing to >50% within 10–12 years after injury [1]. Current PTOA therapies utilized in humans include arthroscopic surgical techniques such as repair/reconstruction, tissue debridement, microfracture and matrix-induced chondrocyte or autologous chondrocyte implantation. Less invasive treatments include injections of hyaluronan, platelet-rich plasma, bone marrow aspirate concentrate, and steroid injections [[3], [4], [5], [6], [7]]. However, the true therapeutic value of these treatments is still not established. In many cases, the injury results in joint degeneration, which progresses to clinical PTOA that may eventually require joint replacement surgery [[3], [4], [5],^8^].

PTOA is also a prevalent and debilitating musculoskeletal condition in horses, compromising their physical and work performance and resulting in economic losses to the horse industry [8]. Racehorses commonly experience injuries and higher levels of joint wear and tear on their joints, versus non-racehorses; as such, they have a very high prevalence of PTOA, akin to human athletes [8,9]. As horses and humans have similarities in their cartilaginous surfaces (similar cellular and molecular composition, mechanics, and anatomy) [9], this research is highly translatable to humans. Successful treatments in horse studies can be useful for predicting human clinical efficacy.

In recent years, cellular therapies using adult multipotent cells have received attention and efforts have been made in preclinical and clinical studies to evaluate their therapeutic potential in cartilage repair [5]. Mesenchymal stromal cells (MSCs) are the most common cell type explored for treating joint injury and disease and can be harvested from various tissues, e.g., bone marrow, adipose tissue, and placenta [[10], [11], [12], [13]]. The initial motivation for using MSCs was their ability to differentiate into musculoskeletal cell types (osteoblasts, chondrocytes, adipocytes). However, it has become increasingly apparent that their differentiation into a stable articular cartilage cell type is limited [4,14]. Following these observations, research shifted from MSC's ability to regenerate cartilage to their immunomodulatory effects. MSCs have since been shown to influence the microenvironment by secreting trophic factors, which can modulate the local immune response by inhibiting the proliferation of immune cells [15], downregulating inflammatory and catabolic processes, recruiting endogenous repair cells [13,[15], [16], [17]], and modulating metabolism via bioenergetic exchange and/or mitochondrial transfer [18]. Subsequently, a body of research has emerged suggesting that the therapeutic effect of MSC preparations is primarily through paracrine stimulation of the native tissues' cells to reduce inflammation and enhance repair rather than directly inducing the repair of cartilage defects [14,19]. As such, an increasing importance is being placed on understanding the secretome of cellular therapeutics [9].

Articular cartilage progenitor cells (ACPs) have generated interest as an alternative cell source to MSCs for application in the development and regeneration of cartilage [[20], [21], [22], [23], [24], [25], [26], [27], [28], [29]]. They have been isolated and extensively characterized from bovine [20,22], human [[25], [26], [27]], and equine [23] articular cartilage. In vitro, they exhibit a stable chondrogenic phenotype when differentiated into cartilage in pellet cultures. They also have a reduced potential for terminal differentiation towards a less desirable hypertrophic chondrocyte phenotype, which is the fate of MSCs in the same assay [27]. Furthermore, ACPs exhibit high colony-forming efficiency and can proliferate in culture for numerous population doublings (up to 75) without telomere shortening [22,23]. Importantly, they are cloneable, enabling the selection of single-cell-derived clones with a highly predictable differentiation phenotype. This contrasts with MSCs, which are challenging to clone and expand without phenotypic drift [25,30,31]. The significant advantages of retention of a stable chondrogenic phenotype over extended population doublings, along with the potential for generating sizeable cell numbers, make ACPs uniquely suited as a chondrocyte cell source compared with MSCs for cartilage tissue engineering applications [23,31,32].

Regardless of cell type or source, successful cellular therapies should not only be able to slow cartilage degeneration and initiate repair but also address synovial inflammation, joint pain, stiffness, and swelling attributed to OA [33,34]. If ACPs are also shown to act as immune modulators, they would be a promising candidate for PTOA therapies. As such, our objectives were to: (1) compare the immunomodulatory secretory phenotypes of ACPs with MSCs in vitro using stimulated macrophage cytokine profiling; (2) determine the efficacy of ACPs and MSCs in reducing clinical signs of OA (via behavior/mobility and gait analysis); and (3) evaluate the effects of ACPs and MSCs on joint pathology. We hypothesized that injected ACPs would be more effective at slowing the progression of PTOA in the DMM mouse model than either the vehicle control (Lactated Ringer's solution (LRS)) or MSCs. Given the clonal expandability and phenotypic stability of ACPs, compared to the typically heterogeneous nature of MSC populations, this study was designed as a feasibility-oriented comparison reflecting practical application rather than a strictly controlled equivalence between cell preparations.

## 2 Materials/methods

### 2.1 In vitro phase

#### 2.1.1 Bone marrow collection/processing (MSC generation)

Bone marrow was aseptically collected from the sternebrae of three skeletally mature mixed breed horses, euthanized at Colorado State University's (CSU) Veterinary Teaching Hospital (VTH) under Institutional Animals Care and Use Committee (IACUC) #1714 and #5672 for clinical pathologies not related to joint problems (Fig. 1). At euthanasia, a stab incision was created on the midline, and a Jamshidi needle was advanced into the medullary cavity. Bone marrow aspirate (BMA) was collected into a 60 cc syringe with a 5:1 ratio (BMA:Heparin 1000IU/mL). BMA was plated in T-75 cell culture flasks and incubated for 5–7 days in complete growth media (Dulbecco's Modified Eagle's Medium (DMEM), glucose (1000 mg/L), 10% fetal bovine serum (FBS), penicillin (100 U/mL), streptomycin (100 μg/mL), HEPES (1 M)) to isolate MSCs. MSCs were expanded to confluency and then frozen in freeze media (95% FBS and 5% DMSO) before use in subsequent assays.

**Fig. 1:**
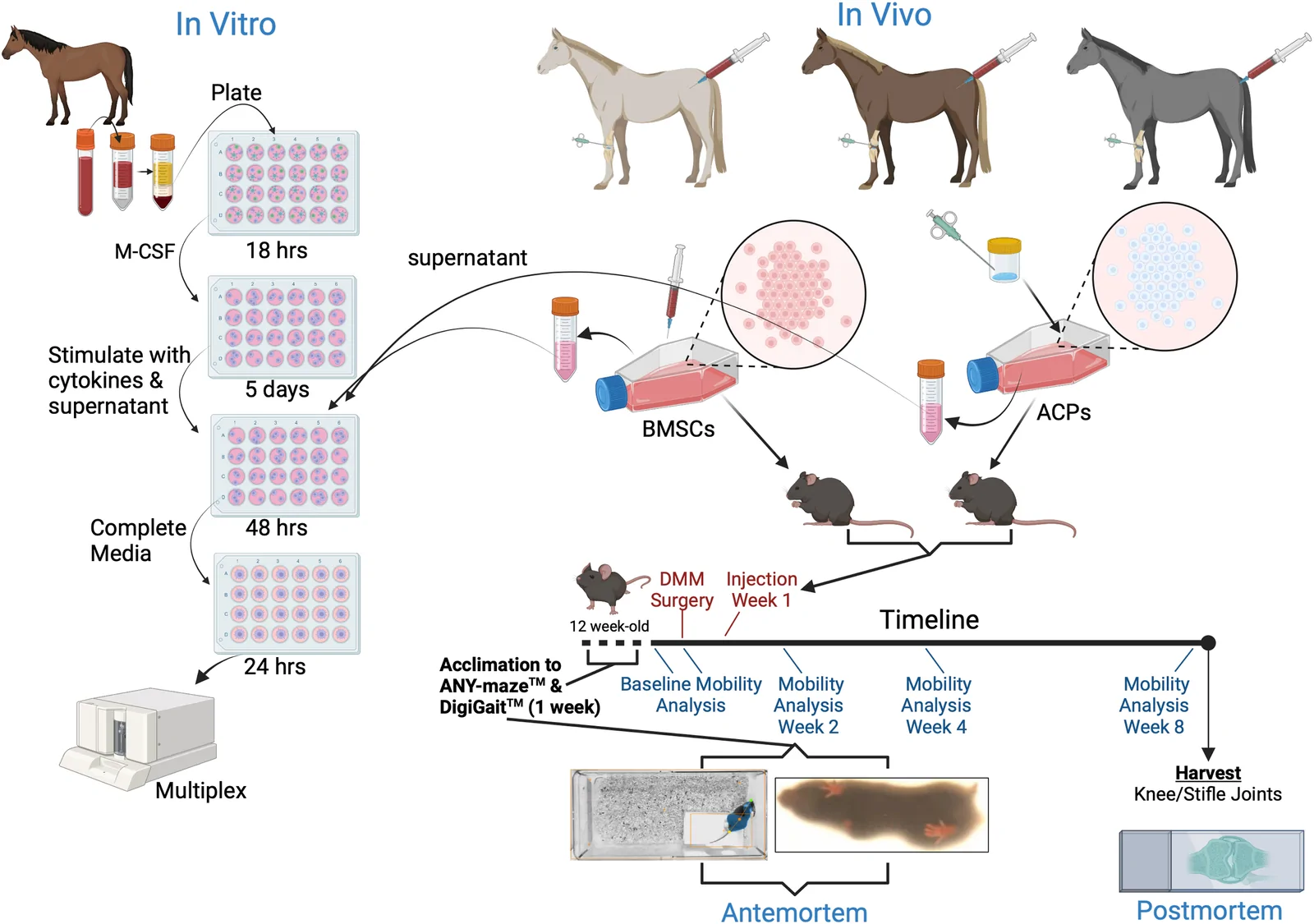
**Schematic representing procedures and methods of this study**. In the in vitro phase, equine blood was collected and monocytes were isolated, plated, and stimulated to differentiate into macrophages. After differentiation, macrophages were treated with supernatant from BMSCs (aka MSCs), ACPs, and inflammatory cytokines. The cells were then washed and allowed to grow for another 24 h; the supernatant was then measured for inflammatory cytokines by multiplex. In the in vivo phase, the isolated MSCs and ACPs from each of three horses (or Lactated Ringer's solution, vehicle) were injected intraarticularly into mice stifles that had received a destabilization of the medial meniscus surgery 1 week before injection. Animal behavior/mobility and gait were monitored until week eight post-injury, at which time mice were euthanized and joints were examined histologically. Acronyms: BMSCs (Bone marrow-derived mesenchymal stromal cells), MSC (mesenchymal stromal cells), ACPs (articular cartilage progenitor cells), DMM (destabilization of medial meniscus), M-CSF (Macrophage colony-stimulating factor). Image created using Biorender.com.

#### 2.1.2 Cartilage collection/processing (ACP generation)

Healthy articular cartilage was aseptically collected from the medial femoral trochlea of the stifle joint of the same three donor horses (Fig. 1). The cartilage was serially digested in pronase (10 mg/mL, Sigma-Aldrich, SKU#11459643001) for 1 h at 37 °C and 5% CO_2_ and then in collagenase type II (1300 IU/mL, Worthington Biochem, Cat.#LS004174) for 3 h at 37 °C and 5% CO_2_. Six-well plates were coated with fibronectin (10 μg/mL, Sigma-Aldrich, SKU#341631) overnight at 4°C^25^, and cells were plated at 1000 cells/mL in complete media + Ascorbate-2-Phosphate 5 mL (10 mM), fibroblast-growth-factor (FGF)-2 (5 ng/mL, PeproTech, Cat.#100-18 B), and human-transforming-growth-factor-beta-1 (0.5 ng/mL, PeproTech, Cat.#100-21C) to isolate articular-chondroprogenitor cells. Colony-forming cells were isolated using cloning rings, expanded in 12-well plates, and then T-75 flasks. At confluency, cells were removed and frozen.

#### 2.1.3 Generation of conditioned media

ACPs and BMSCs were rapidly thawed, washed, and plated on 12-well plates in 1 mL complete DMEM at 100,000 cells/mL for 24 h at 37 °C and 5% CO_2_. Media was changed to DMEM + penicillin/streptomycin and incubated for 24 h (Fig. 1). Media was collected and stored at −20 °C. Monocyte-derived macrophages were used to assess the immunomodulatory effects of conditioned media (see Fig. 1 and Supplemental Materials for preparation and stimulation methods).

#### 2.1.4 Multiplex immunoassay for cytokine quantification

A fluorescent bead-based multiplex assay (Milliplex MAP Equine Cytokine/Chemokine Magnetic Beads Multiplex Assay, Millipore Sigma, SKU# EQCYTMG-93KPX23), run according to the manufacturer's protocol in duplicate for each of the three horses, was used to quantify the concentrations of 23 analytes (Eotaxin/CCL11, FGF-2. Fractalkine/CS3CL1, granulocyte colony-stimulating factor (G-CSF), granulocyte-macrophage colony-stimulating factor (GM-CSF), growth regulated oncogene (GRO), interferons (IFN), interleukin (IL)-1α, IL-1β, IL-2, IL-4, IL-5, IL-6, IL-8/CXCL8, IL-10, IL-12 (p70), IL-13, IL-17a, IL-18, IP-10, monocyte chemoattractant protein (MCP-1/CCL2), regulated on activation normal T-cell expressed and secreted (RANTES/CCL5), and TNF) in cell culture supernatants.

### 2.2 In vivo phase

#### 2.2.1 Animals

All animal procedures were approved by CSU IACUC #2778 and were performed following the NIH Guide for the Care and Use of Laboratory Animals. Seventy male C57/6 J mice (Jackson Laboratories) were utilized in this study. Mice arrived at 11 weeks of age and underwent a 1-week acclimation and training period prior to injury at 12 weeks [35] (Fig. 1). For this study, male mice were utilized as they develop PTOA to a higher severity than female mice in the destabilization of the medial meniscus (DMM) model [35,36]. Thus, sex was not a variable in this study. Mice were co-housed in solid-bottom cages in groups of five and allowed ad libitum water and standard rodent chow. A veterinarian assessed all animals daily, including mild, transient post-procedure edema; body weight was monitored weekly. Animals were kept at 20–25 °C with a 12-12 h Light-dark cycle and 30–70% humidity. All animals received a DMM surgery of the right hind stifle and an intraarticular injection (for detailed methods, see Supplementary Material) (Fig. 1). Mice were euthanized via CO_2_ inhalation, confirmed by exsanguination and cervical dislocation, at eight weeks post-injury (mid-stage PTOA) [35,37,38].

#### 2.2.2 Therapeutic injections

Mice from a single shipment were randomly placed into cages of 5 mice each. Each cage was then randomly selected to receive LRS or a particular treatment group from one of the three horses (Fig. 1). At one-week post-injury, mice were anesthetized, placed supine, and the previously injured knee was aseptically prepared. Mice were divided into three treatment groups receiving either LRS (n = 10), ACPs (50,000 cells) (n = 30; 10 animals per horse), or MSCs (50,000 cells) (n = 30; 10 animals per horse). Treatments were provided in 10 μL of LRS using a 27-gauge insulin syringe placed just medially to the patellar tendon.

#### 2.2.3 *ANY-maze*^*TM*^ cage monitoring

Animal behavior/mobility was performed with the *ANY-maze*^*TM*^ software (Wood Dale, IL) utilizing a 10-min data collection period in an open-field, free movement scenario. The cage setup utilized each animal's home cage and a square residential security hut [35,38] (Supplementary Fig. 2D). Before the DMM injury, animals were acclimated twice to ensure familiarity with the setup; two baseline measurements were then performed on separate days (baselines were averaged for analyses). Cage monitoring occurred at weeks 2, 4, and 8 post-injury; for every non-collection week, a 2-min solo maintenance procedure was performed. Acclimation, maintenance, and data collection were performed during the same timeframe (6–10 a.m.) and involved the same handlers. The ANY-maze™ data analysis focused on the six most clinically relevant OA parameters [distance traveled, time mobile not spent in resident environmental enrichment hut (visible time mobile), average speed, top of hut entries, time in resident environmental enrichment hut (time in hut), and freezing time] [35,38] (Fig. 3) (see Table 1 for in-depth descriptions of parameters). Tests were excluded if the computer failed to track accurately (Supplementary Table 2).

**Fig. 2:**
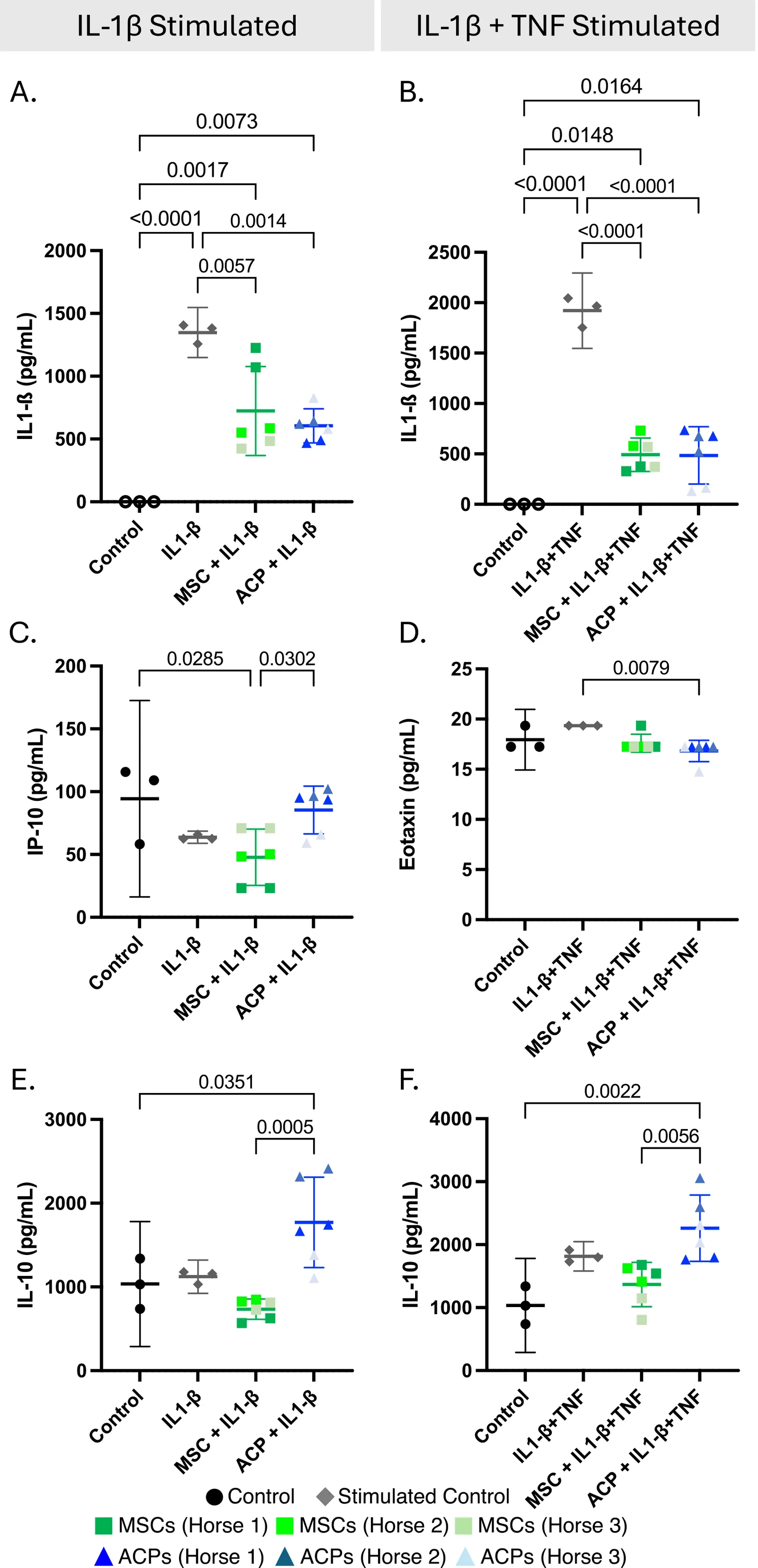
**Macrophages Stimulated with Pro-inflammatory Cytokines.** Multiplex immunoassay of equine monocyte-derived macrophage cell culture supernatant measuring inflammatory cytokine response after IL-1β or IL-1β + (tumor necrosis factor) TNF stimulation. Controls are unstimulated macrophages. Unmeasurable values are reported as zero and signified as an empty shape. **A**. **IL-1β levels** after IL-1β stimulation [(Control vs. IL1β: p < 0.0001, control mean 0, 95% CI 0-0; IL1β mean 1348, 95% CI 1150–1547) (Control vs. MSC + IL1β: p = 0.0017, MSC + IL1β mean 723.5, 95% CI 369.9–1077) (Control vs. ACP + IL1β p = 0.0073, ACP + IL1β mean 605.3, 95% CI 469.7–741) (IL1β vs. MSC + IL1β p = 0.0057) (IL1β vs. ACP + IL1β p = 0.0014)]. **B**. **IL-1β levels** after IL-1β + TNF stimulation [(Control vs. IL1β+TNF: p < 0.0001, control mean 0, 95% CI 0-0; IL1β+TNF mean 1922, 95% CI 1548–2295) (Control vs. MSC + IL1β+TNF: p = 0.0148, MSC + IL1β+TNF mean 492.2, 95% CI 326.4–658.1) (Control vs. ACP + IL1β+TNF: p = 0.0164, ACP + IL1β+TNF mean 485.1, 95% CI 201–769.3) (IL1β+TNF vs. MSC + IL1β+TNF: p < 0.0001) (IL1β+TNF vs. ACP + IL1β+TNF: p < 0.0001)]. **C. IP-10 levels** after IL-1β stimulation [(Control vs. MSC + IL1-β: p = 0.0285, control mean 94.41, 95% CI 16.28–172.5; MSC + IL1-β mean 47.8, 95% CI 25.38–70.21) (MSC + IL1-β vs. ACP + IL1-β: p = 0.0302, ACP + IL1-β mean 85.49, 95% CI 66.48–104.5)] **D**. **Eotaxin (CCL11) levels** after IL-1β + TNF stimulation [(IL1β+TNF vs. ACP + IL1β+TNF: p = 0.0079, IL1β+TNF mean 19.35, 95% CI 19.35–19.35; ACP + IL1β+TNF mean 16.83, 95% CI 15.76–17.89)]. **E**. **IL-10 levels** after IL-1β stimulation [(Control vs. ACP + IL1-β: p = 0.0351, control mean 1036, 95% CI 289.8–1781; ACP + IL-1β mean 1772, 95% CI 1232–2311) (MSC + IL1β vs. ACP + IL1β: p = 0.0005, MSC + IL1β mean 734.6, 95% CI 612.7–856.4)]. **F**. **IL-10 levels** after IL-1β + TNF stimulation [(Control vs. ACP + IL1-β+TNF: p = 0.0022, control mean 1036, 95% CI 289.8–1781; ACP + IL1-β+TNF mean 2261, 95% CI 1733–2788) (MSC + IL1-β+TNF vs. ACP + IL1-β+TNF: p = 0.0056, MSC + IL1-β+TNF mean 1368, 95% CI 1017–1720)]. **Statistics**: Line at mean; error bars mean ± 95% CI; calculated with an ordinary one-way ANOVA & Tukey's multiple comparisons test with Prism GraphPad version 10. For all statistics, see Supplementary Tables 4 and 5**Acronyms**: IL-1β (interleukin 1 beta), TNF (tumor necrosis factor), IP-10 (interferon *γ*-induced protein 10), interleukin 10 (IL-10), CCL11 (C–C motif chemokine 11), MSCs (mesenchymal stromal cells), ACPs (articular cartilage progenitor cells).

**Fig. 3:**
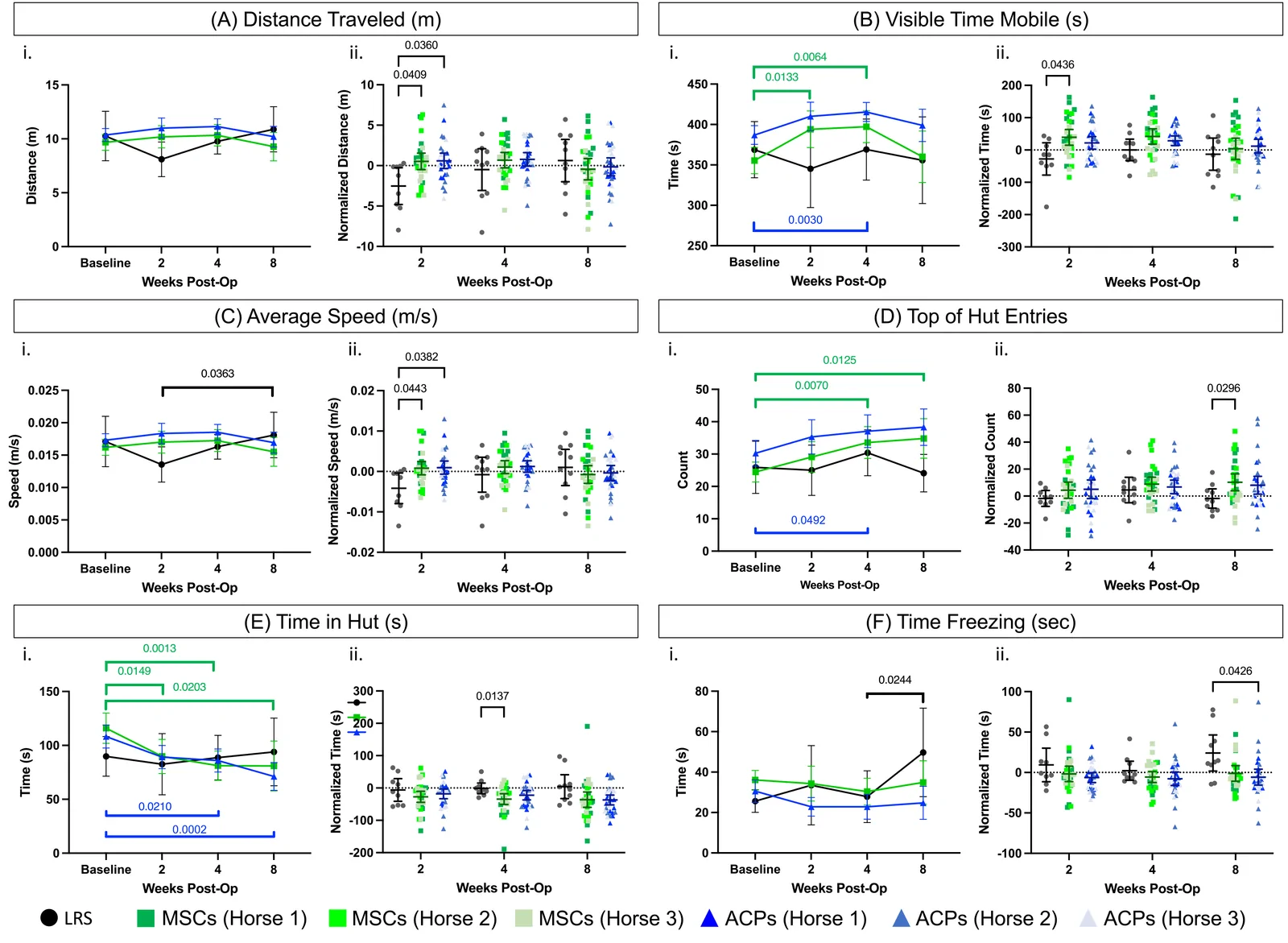
**ANY-maze Open Field Overhead Cage Monitoring.** Analysis of animal behavior and mobility over time and between treatment groups. Data was collected with two (averaged) baselines prior to injury and at weeks 2, 4, and 8 post-injury. The left plots (**i.**) show longitudinal (over time) raw measures. The right plots (**ii.**) show comparisons between treatments at weeks 2, 4, and 8 normalized to baseline (i.e., the difference from the averaged baseline measure for each animal). **A. Distance Traveled** (meters); **B. Visible Time Mobile** (seconds); **C. Average Speed** (meters/second); **D. Top of Hut Entries** (count); **E. Hut Time** (seconds); **F. Freezing Time** (seconds). **Statistics:** line at mean, error bars mean ± 95% CI, calculated with a linear mixed model, with multiple comparisons in Prism GraphPad version 10, p values, means, and 95% CIs can be found in Table 2, additional statistics see Supplementary Table 6. **Acronyms:** MSCs (mesenchymal stromal cells), ACPs (articular cartilage progenitor cells), LRS (Lactated ringer's solution) vehicle control.

**Table 1:**
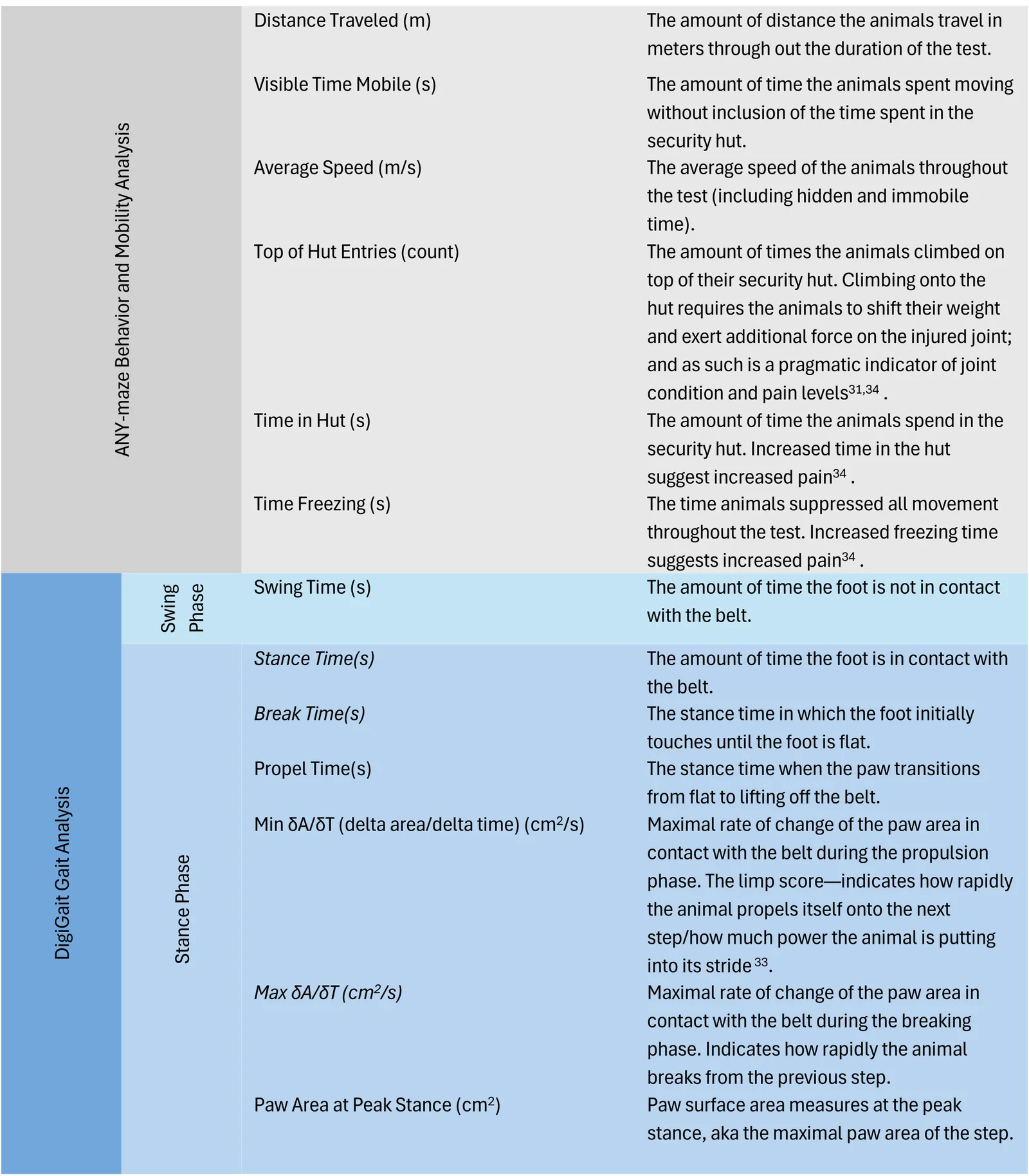
ANY-Maze™ and DigiGait™ parameter Descriptions.

#### 2.2.4 DigiGait™ gait analysis

Mouse gait outcomes were measured via compulsory treadmill running with the DigiGait™ analysis system (Mouse Specifics, Inc., Framingham, MA). Animals were run on the treadmill at both flat and 10° incline at a speed of 40 cm/s in triplicate (triplicates were averaged for analysis) for five or more full steps. A camera below the belt recorded footstep patterns for gait analysis (Fig. 4A). In the week before the DMM injury, animals were trained to run, acclimated twice, and two days of baseline running were collected (baselines were averaged for analyses). Post-injury data were collected at weeks 2, 4, and 8. Mice were maintained under the same conditions every non-data collection week. Acclimation, maintenance, and data collection were performed during the same timeframe (12-4 pm) and involved the same handlers. Data processing was performed using the DigiGait™ software. Evaluated parameters for the right hind leg included swing speed, break time, propel time, stance time, stride length, stance width, paw area at peak stance, min δA/δT, max δA/δT, and gait symmetry (see Table 1 for in-depth descriptions of parameters); these outcomes have been previously reported for DigiGait™ [37]^,^ [39]. Single runs were excluded if stride length or stride length %CV values were physiologically implausible, deviated substantially from same-day runs, or had a %CV > 20. Animals were also excluded if they were unable to complete treadmill runs at that timepoint and future timepoints (Supplementary Table 3).

**Fig. 4:**
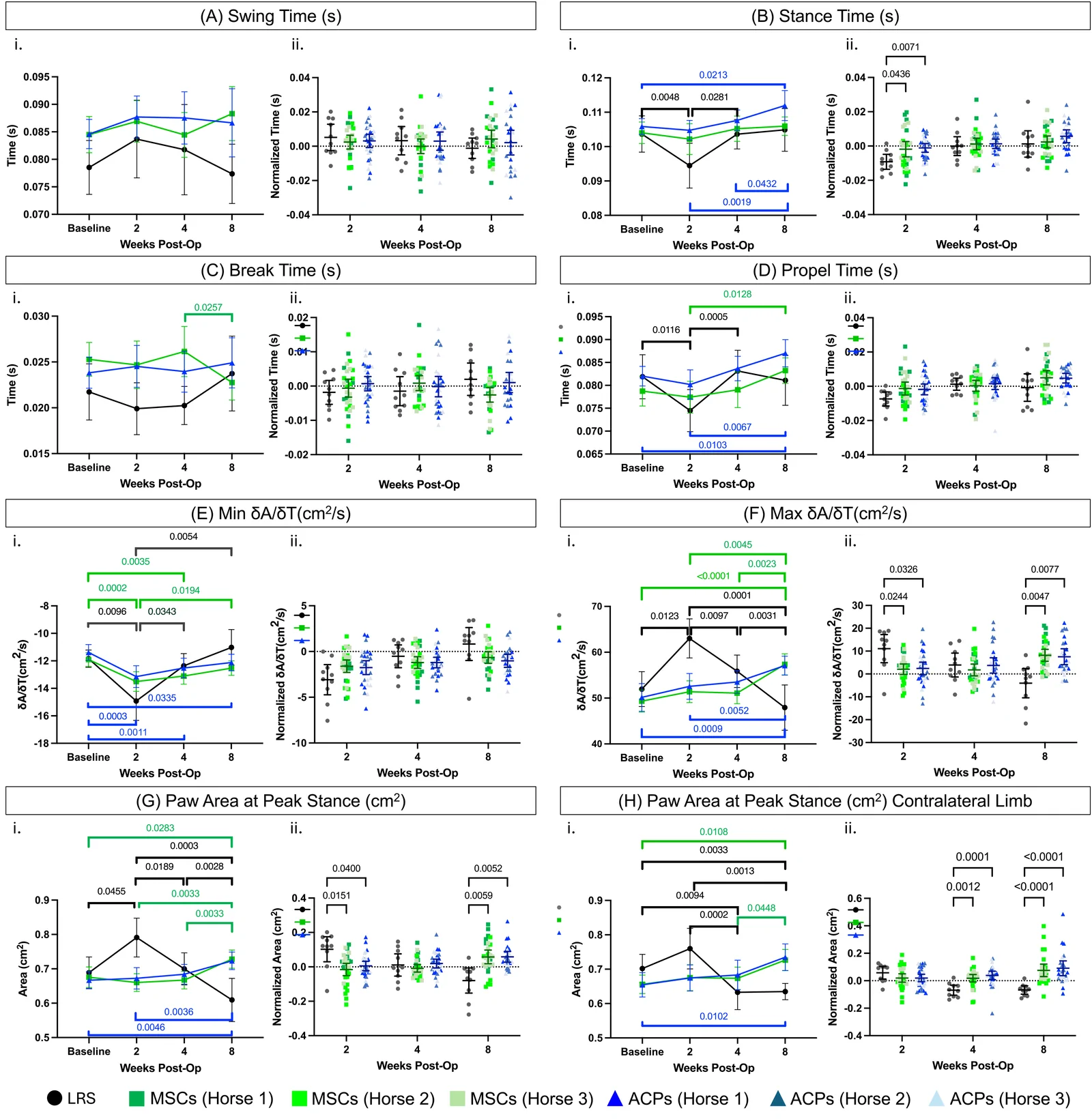
**DigiGait Gait Measures. Analysis of gait over time and between treatment groups. Data was collected with two (averaged) baselines prior to injury and at weeks 2, 4, and 8 post-injury. The left plots (i.) show longitudinal (over time) raw measures. The right plots (ii.) show comparisons between treatments at weeks 2, 4, and 8 normalized to baseline (i.e., the difference from the averaged baseline measure for each animal). (A**–**D) Gait Time Measures. A. Swing Time (seconds); B. Stance Time (seconds); C. Break Time (seconds) (see**Supplementary Fig. 4**(F), breaking phase); D. Propel Time (seconds); (see**Supplementary Fig. 4 (**G**) propelling phase). (**E-H**) **Paw Area Measures. E. Min δA/δT** (delta area/delta time) (centimeters per second squared) (limp score); **F. Max δA/δT** (centimeters per second squared; **G. Paw Area at Peak Stance** (centimeters per second squared); **H. Paw Area at Peak Stance of the Contralateral Limb** (centimeters per second squared); **Statistics:** line at mean, error bars mean ± 95% CI, calculated with a linear mixed model, with multiple comparisons in Prism GraphPad version 10, p values, means, and 95% CIs can be found in Table 3 for additional statistics see Supplementary Table 7. **Acronyms:** MSCs (mesenchymal stromal cells), ACPs (articular cartilage progenitor cells), LRS (Lactated ringer's solution) vehicle control.

#### 2.2.5 Histologic assessment of knee/stifle joints

Following euthanasia, hind limbs were removed and fixed in 10% neutral buffered formalin for 24 h. After fixation, limbs were decalcified in 12.5% ethylenediamine tetra-acetic acid (EDTA; pH 7.2–7.4 for approximately 3.5 weeks, changing the EDTA solution every 2–3 days). Joints were embedded in paraffin; 5 μm coronal sections were taken from the center of the medial tibial plateau and stained with toluidine blue [2,35,37,38]. Joints were scored with a modified protocol based on the Haubruck et al. scoring system (Supplementary Table 1) by a blinded veterinary pathologist (KSS) [40].

### 2.3 Statistical analysis

Sample size determinations for the in vivo study were calculated with histological assessment of OA as the primary outcome based on prior experiments using the DMM model (for full details, see Supplemental Material) [35]. Donor (horse) was evaluated as a variable and did not significantly influence outcomes. All statistics were conducted in GraphPad Prism v10 and passed normality. Statistical significance is denoted as p < 0.05. Cytokine panels were analyzed with an ordinary one-way ANOVA and Tukey's multiple comparisons tests [35]. *ANY-maze*™ and DigiGait™ were analyzed with a Mixed-effects model with the Geisser-Greenhouse correction and a Tukey's multiple comparisons test. Histological scoring was analyzed with a Kruskal-Wallis test with Dunnett's Multiple comparisons.

## 3 Results

### 3.1 In vitro analysis of cytokines

***Impact of ACP and MSC treatments on cytokine secretion from IL-1β/TNF-activated macrophages.*** In our multiplex immunoassay, there were measurable concentrations for 11 out of 23 cytokines (FGF, Eotaxin, GM-CSF, IL-1β, IL17-A, IL-8, IP-10, GRO, IL-10, TNF, and RANTES). Notably, both the ACP and MSC supernatant treatment groups showed reduced IL-1β production compared to the positive control (cytokine-stimulated macrophages) under both stimulation conditions (IL-1β and IL-1β+TNF) (Fig. 2A and B). For macrophages stimulated with IL-1β+TNF and treated with the ACP supernatant, there was reduced production of pro-inflammatory cytokine Eotaxin (CCL11), from stimulated controls (Fig. 2D). IP-10, for IL-1β stimulated cells, was increased in the ACP treated cells compared to MSCs (Fig. 2C). Additionally, under both stimulation conditions, macrophages treated with ACP supernatant had increased levels of the anti-inflammatory cytokine IL-10 compared to MSC supernatant-treated cells (Fig. 2B). For additional graphs and statistics displaying all measurable analytes, see Supplementary Fig. 1 and Supplementary Tables 4–5.

### 3.2 In vivo functional outcomes

#### 3.2.1 Behavior and mobility analysis

Over time differences: MSC and ACP treatment groups showed increased mobility across time. Specifically, both treatment groups had increases in mobile time(s) from baseline (Fig. 3Bi) and increases in top-of-hut entries from baseline (Fig. 3Di). Additionally, both treatment groups spent less time in their security hut than baseline (Fig. 3Ei). We also observed changes across time in our LRS-control group (Fig. 3C and F), with decreased average speed (m/s) between weeks 2 and 8 (Fig. 3Ci) and increased time(s) freezing between weeks 4 and 8 (Fig. 3Fi) (Table 2).

**Table 2:**
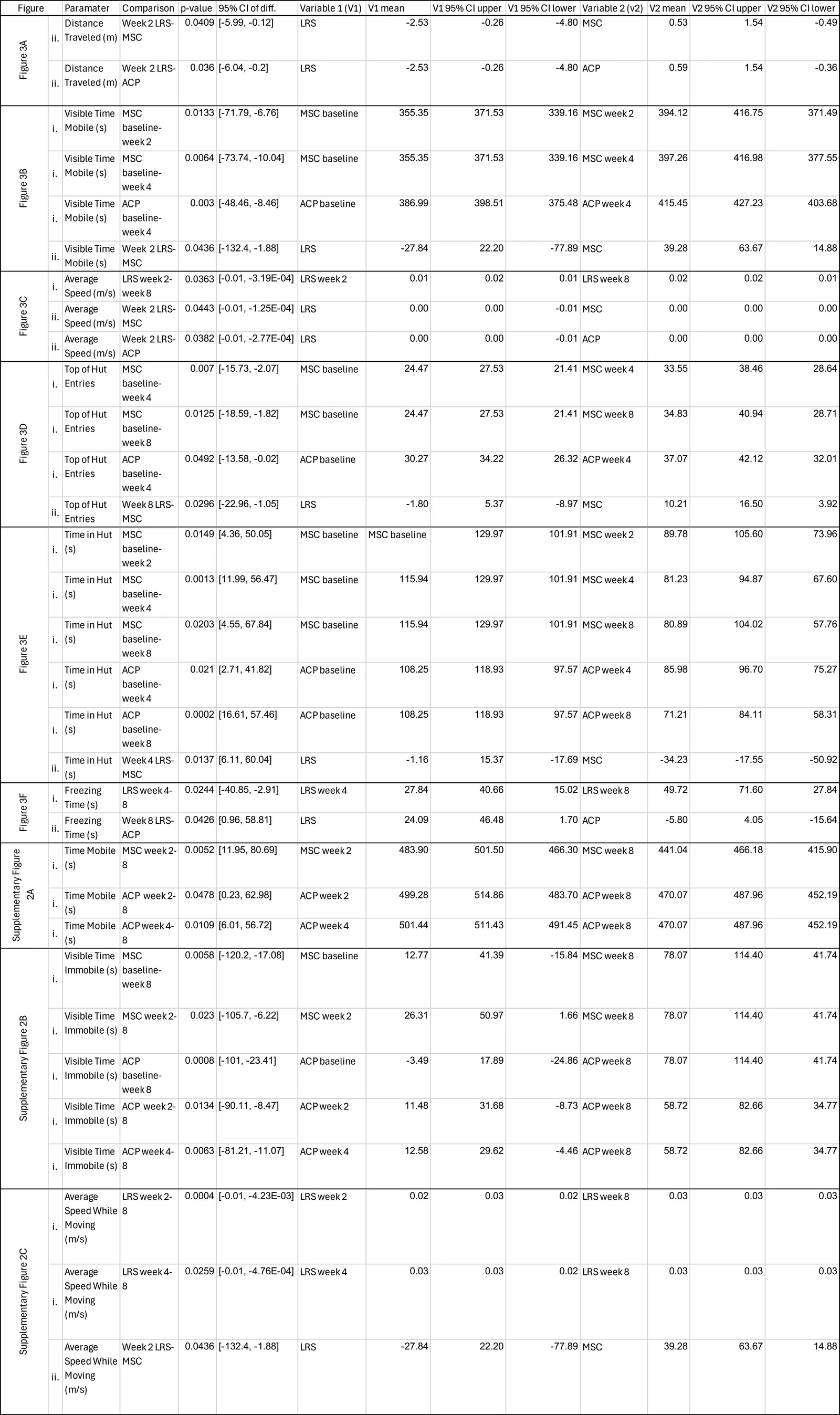
ANY-Maze™ overhead cage monitoring parameters Statistics.

Differences between groups: We observed short-term (week 2) beneficial effects that allowed the treated groups to maintain baseline behavior/mobility levels while the LRS-control animals declined in the initial phase of injury (Fig. 3). Specifically, LRS-controls decreased significantly (compared to both treatment groups unless noted) for distance traveled(m) (Fig. 3Aii), visible time mobile(s) (MSCs only) (Fig. 3Bii), and average speed (Fig. 3Cii). ACP and MSC-treatment groups also demonstrated longer-term (weeks 4–8) effects on mobility. Specifically, the MSC-treated group had decreased hut time at week 4 (Fig. 3Eii) and increased top-of-hut entries at week 8 (Fig. 3Dii) than the LRS-control group. The ACP-treated group had decreased time freezing at week 8 compared to LRS-controls (Fig. 3Fii) (Table 2).

***Gait Analysis:*** The DigiGait™ system tracks the animal's stride and divides it into two main phases: swing and stance (Table 1 & Supplementary Fig. 4E). The stance phase has additional sub-phases: brake, peak stance, and propel (Supplementary Fig. 4F–H). Overall, parameters for the stance component demonstrated injury and treatment-related changes (Fig. 4B–H), while the swing phase of the step was statistically unimpacted (Fig. 4A).

Over time differences: LRS-control animals exhibited a pronounced shift in gait compensation following DMM injury, initially adopting one compensatory strategy that transitioned to an opposing pattern in later stages. Initially (week 2) the LRS-control animals decreased their step propulsion (Min *δA/δT*), but by week 8, they had increased step propulsion (Fig. 4Ei); conversely, they had increased foot slamming in breaking (Max *δA/δT)* and greater paw area at week 2 (Supplementary Fig. 4C), but by week 8, they were barely breaking and barely putting their foot down on the belt (Supplementary Fig. 4D and 4Fi-Gi). The gait changes in the ACP and MSC-treatment groups occurred more gradually than the LRS-controls and were observed in the later phase of the injury (weeks 4–8), where the animals were starting to demonstrate some of the stride changes seen in the LRS-control animals in the initial stage injury (Fig. 4Bi, Di-Gi) (Table 3).

**Table 3:**
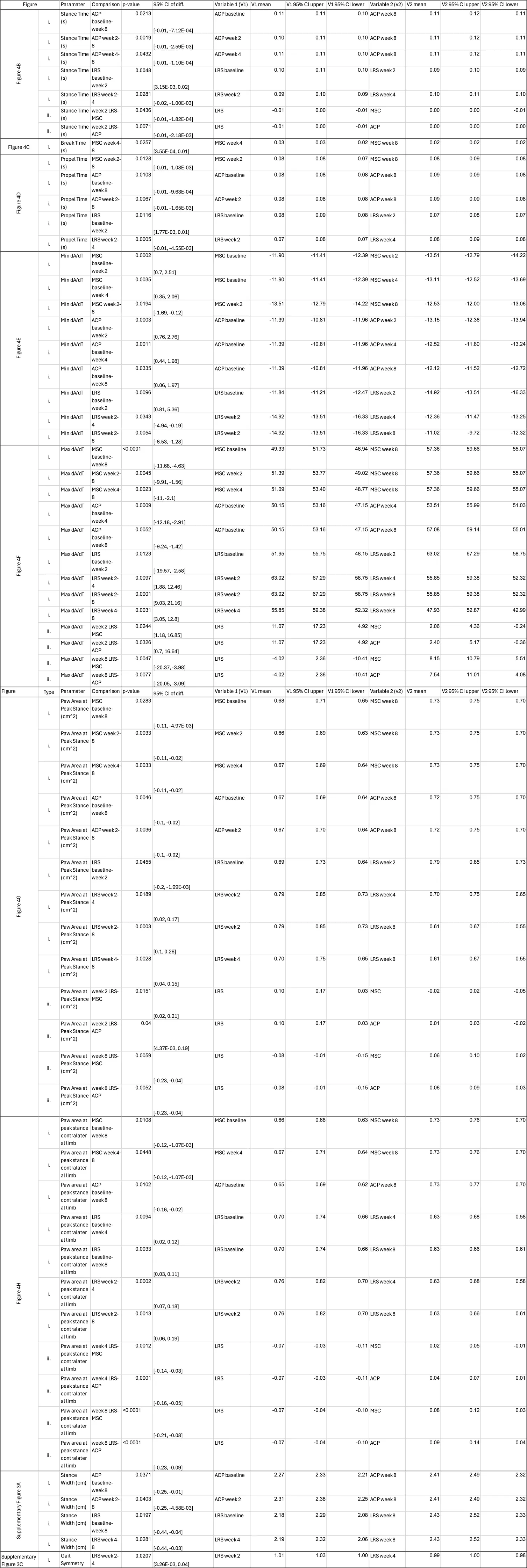
DigiGait™ gait parameters Statistics.

Differences between groups: The ACP and MSC treatment groups exhibited gait protection both in the initial injury phase and in the later phase. At week 2, the LRS-controls were significantly more impacted than either of the treatment groups—which more closely maintained the baseline measures. Specifically, the LRS-control animals had increased paw area (Fig. 4Bii), a heavier breaking phase (Fig. 4Fii), and increased paw area (Fig. 4Gii). At week 8, the LRS-control group shifted to minimal breaking (Fig. 4Fii) and toe-touch running (Fig. 4Eii), where both treatment groups exhibited heavier breaking and increased surface area. We also observed compensatory limb effects at week 4 for the LRS-control groups and 8 for all three groups (Fig. 4Hii) (Table 3).

#### 3.2.2 Evaluation of knee joint pathology

Medial compartment joint scores demonstrated significant improvement with the ACP treatment over the MSC treatment (MSC-ACP p = 0.0009, mean 31.56, 95% CI 24.52–38.61, ACP 17.26 mean, 95% CI 13.53–20.99) and LRS-controls (LRS-ACP p = 0.0019, LRS mean 34.06, 95% CI 27.72–40.39) (Fig. 5D). Structurally, the ACP-treated animals exhibited less variability in proteoglycan staining and a smoother surface across the entire articular surface of the tibia and femur (Fig. 5C) than the MSC-treated and LRS-controls. MSC-treated and LRS-control animals exhibited greater loss of proteoglycan, increased synovitis, chondrocyte hypertrophy or loss, and fibrillation (Fig. 5A–B) than ACP-treated animals.

**Fig. 5:**
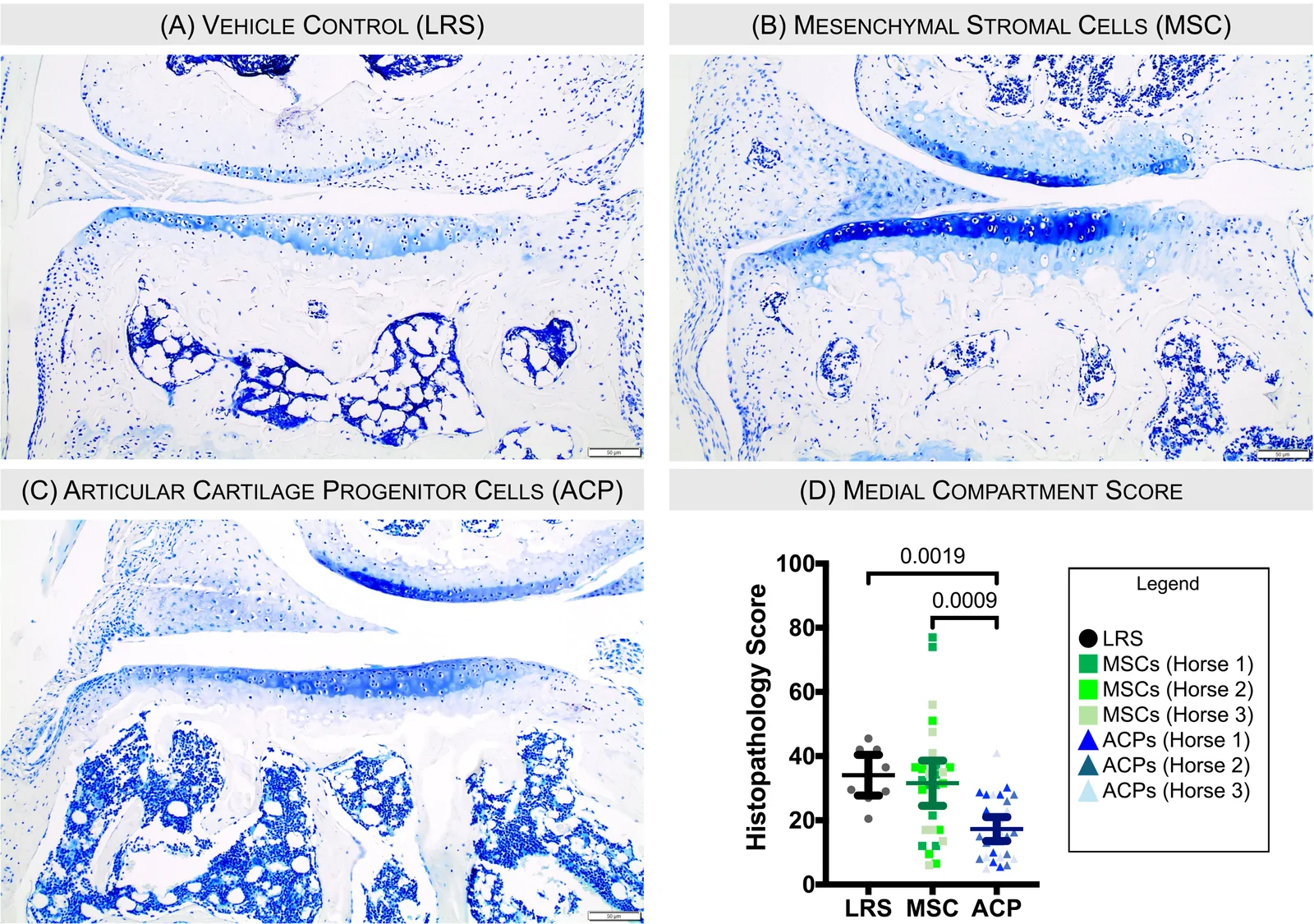
**Histopathology Scoring of the Medial Compartment of the Right Hind Limb at 8 weeks post DMM.** (**A-C**) Representative images of median medial compartment score by treatment showing the articular cartilage surface stained with toluidine blue. **A.** LRS vehicle control, representative image scored 42**. B.** MSC representative image scored 31. **C.** ACP representative image scored 5.5. **D.** Histological scoring, sum of all components of the medial compartment of the joint (see Supplementary Table 1 for all scored parameters). See Supplemental Fig. 5 for scoring by location within the medial compartment. **Statistics**, line at mean, error bars mean ± 95% CI, one-way-ANOVA Kruskal-Wallis test—Prism GraphPad v10 for additional statistics see Supplementary Table 8. **Acronyms**: DMM (destabilization of medial meniscus), MSCs (mesenchymal stromal cells), ACPs (articular cartilage progenitor cells), LRS (Lactated ringer's solution) vehicle control.

## 4 Discussion

Findings from this study support our hypothesis that injected ACPs are more effective at slowing the progression of PTOA in the DMM mouse model than either the LRS-control or MSCs. Both cellular injections improved functional outcomes in behavioral/mobility and gait compared with controls and effectively maintained joint integrity, thereby slowing the progression of PTOA. These improvements may be associated with the enhanced immunomodulatory profile observed in ACP secretomes, although further research is required to support this directly.

Increasing importance is being placed on understanding the role of the secretome for OA cellular therapeutics [9]. Recent studies with MSC therapeutics demonstrate that paracrine signaling factors produced by stromal or progenitor cells may drive the anti-inflammatory and healing response for successful OA therapeutics [7,9,13]. Additionally, studies have shown that repeated injections of extracellular vesicles—such as exosomes from embryonic MSCs and human adipose-derived stem cells—can reduce joint pathology following DMM injury in mice, suggesting their potential as a therapeutic option [7,41].

Although the immunomodulatory properties of MSCs have been well characterized, and one study examined a mixed cartilage cell population containing ACPs [42], to our knowledge, no studies have investigated the immunomodulatory profile of isolated, clonal-ACPs. Thus, this is the first study to demonstrate that cloned ACPs exhibit a more pronounced anti-inflammatory phenotype than MSCs. Both ACP and MSC secretomes significantly reduced IL-1β in inflamed equine macrophages—a key target given IL-1β′s role in driving cartilage degradation by promoting the release of key catabolic enzymes (MMP-1, MMP-3, MMP-13, and ADAMTS-5) [41]. However, ACP secretomes exhibited superior immunomodulatory effects, markedly decreasing Eotaxin (CCL11) and increasing IL-10 levels beyond MSC-treatment. Unlike previously studied fibronectin-selected ACPs, the ACPs used here were randomly selected clonal populations from each horse [25,30], with consistent responses observed across donors. Overall, the increased anti-inflammatory cytokine environment of the ACP-treated macrophages suggests ACPs may be more competent than MSCs at modulating synovial inflammation; notably, ACPs were as effective as or more effective than MSCs in reducing inflammatory markers for all measured cytokines (except IL-10 under IL-1β stimulation). These findings suggest that for ACPs, like MSCs, the therapeutic benefit is largely paracrine mediated [1,4,11,41].

Importantly, this study also demonstrated improvements in joint pathology. The mouse DMM model provides a mild and reliable progression of OA and is the most common mouse PTOA model to study disease progression and treatments [2,35,38,43]. The medial compartment of the femoro-tibial joint is where the predominant PTOA pathological changes occur after the DMM injury [2]; as such, we focused our histological scoring in this compartment. We assessed changes in joint structure, such as loss of proteoglycan, changes in cartilage cell density, fibrillation or structural modifications, joint infiltration, and synovial hypertrophy [2,35,38]. This study showed that the control and MSC animals had significantly worse OA joint scores than the ACP-treated animals at 8 weeks post-injury. Notably, the ACP treatment demonstrated more protection/preservation of articular cartilage surfaces, whereas degradation was present in control and MSC-treated groups. ACP therapies for OA are still a relatively new area of research, but our results are consistent with published work, particularly regarding joint protection in histology slides. Specifically, two chondral defect studies in different animal models—horse and goat—showed significant improvements with ACP treatments [25,30]. While MSCs have been more extensively researched in various animal models and injury types/OA induction, their structural protection is less reliable, with some studies showing joint protection [7,18,19,35], others showing no protection [44,45], and some increasing joint degradation [46].

Although preserving joint structure is important, patients are often more concerned with reducing pain and improving mobility than with pathology scores alone, and small improvements in pain and functional mobility can be life-changing [35,38,47,48]; highlighting the importance of evaluating functional outcomes. In this study, ACP- and MSC-treated animals maintained pre-injury behavior/mobility and gait more effectively than controls. These findings align with previous PTOA studies in both humans and animals, reporting improved functional outcomes following stromal or progenitor cell therapies. A case study of a 26-year-old male with a severe chondral defect reported improvements in pain, mobility, and joint surface integrity following two intra-articular injections of autologous, culture-expanded adipose-derived MSCs administered six months apart [6]. Similarly, studies evaluating weight-bearing pain in animal models reported that MSC treatments reduced pain-related behavior in a collagen-induced OA mouse model, measured via static-incapacitance [45], while autologous and allogeneic ACPs combined with fibrin improved weight-bearing and flexion-associated pain outcomes in horses with chondral defects, particularly in the allogeneic group [30]. To our knowledge, this is the first study to directly compare ACP and MSC treatments in PTOA while evaluating the functional responses during both the acute and chronic phases of the injury.

It is also important to note that this study utilized xenogeneic cell sources based on prior evidence demonstrating the tolerability and efficacy of xenogeneic MSCs in musculoskeletal applications [44,49], as well as the practical limitations of obtaining sufficient matched murine cell numbers. The absence of an overt inflammatory response following xenogeneic MSC injections in previous OA studies [44], together with the relatively immune-privileged nature of the joint environment [50], supported this approach. However, the translational relevance of this work lies less in the xenogeneic source itself and more in the therapeutic potential of clonally expandable ACPs as regenerative and immunomodulatory cell therapies for OA. Future studies should further investigate the mechanisms underlying ACP-mediated immune modulation and tissue protection.^4944^

As this study focused on early-stage PTOA, longer-term studies are needed to determine whether treatment-specific differences persist or become more pronounced over time. Additionally, a limitation of this study was the lack of direct in vivo characterization of inflammatory mediators and immune cell populations within the joint. Future work would be well-positioned to investigate the immunomodulatory effects of MSCs and ACPs in vivo, including assessing mitochondrial transfer, bioenergetic exchange, and characterizing local immune responses via immunohistochemistry. Additional priorities include identifying high-performing ACP clones and key paracrine factors, assessing sex as a biological variable, as understanding sex-specific responses is critical for developing effective therapeutics—and is a current limitation of this study-and translating these findings to larger animal models, including horses.

In summary, this study demonstrated that ACPs have effective anti-inflammatory secretomes capable of reducing inflammation produced by stimulated macrophages, even at a higher capacity than MSCs. ACP injections significantly slow the progression of joint degradation in the mouse DMM model compared to the vehicle and MSCs. Additionally, both ACP and MSC therapeutic injections significantly helped improve pain and maintain mobility, gait, and function in injured mice over LRS-controls.

## Author contributions

**Heidi Kloser:** Investigation, Methodology, Formal Analysis, Writing – original draft.

**Parvathy Thampi:** Conceptualization, Investigation, Methodology, Formal Analysis, Writing - Review & Editing, Funding Acquisition.

**Lynn Pezzanite:** Investigation, Methodology, Formal Analysis, Writing - Review & Editing.

**Taryn Boxleitner:** Investigation, Methodology.

**Lyndah Chow:** Investigation, Methodology, Formal Analysis.

**Steven Dow:** Methodology.

**Katie J. Sikes:** Investigation, Methodology, Writing - Review & Editing.

**Kelly S. Santangelo:** Investigation, Methodology, Writing - Review & Editing.

**Laurie Goodrich:** Conceptualization, Writing - Review & Editing, Funding Acquisition.

**Brian Johnstone:** Conceptualization, Writing - Review & Editing, Funding Acquisition.

## Role of the funding source

The Hong Kong Jockey Club Equine Welfare Research Foundation provided a portion of the funding for the greater study (grant number _PPF-2021-101,639).

## Conflict of interest

L. Goodrich is a shareholder of eQcell. All other authors have no conflicts of interest.
