# Supplementary material for: Clonal articular cartilage progenitors outperform mesenchymal stromal cells in mitigating post-traumatic osteoarthritis
Source: Osteoarthr Cartil Open. 2026 Sep 25;8(4):100878. doi: 10.1016/j.ocarto.2026.100878 (PMC13683781; doi:10.1016/j.ocarto.2026.100878)
Supplement: Supplementary file 1 [file mmc3.pdf]

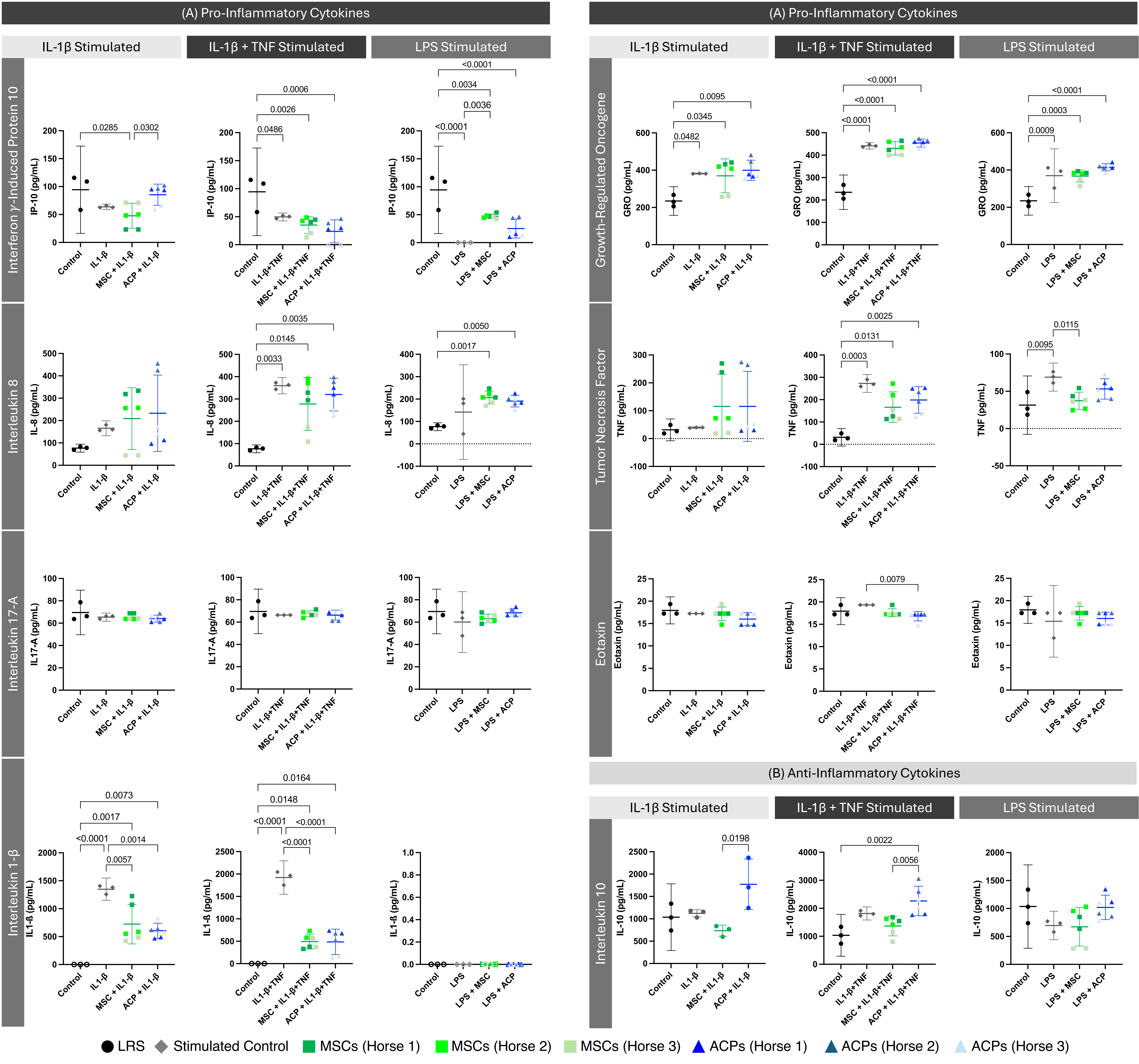
(A) Pro-Inflammatory Cytokines
IL-1β Stimulated
IL-1β + TNF Stimulated
LPS Stimulated
Interferon γ-Induced Protein 10
IP-10 (pg/mL)
Control
IL-1β
MSC + IL-1β
ACP + IL-1β
0.0285
0.0302
IL-1-β+TNF
MSC + IL-1-β+TNF
ACP + IL-1-β+TNF
0.0486
0.0026
0.0006
LPS
LPS + MSC
LPS + ACP
<0.0001
0.0036
0.0034
<0.0001
Interleukin 8
IL-8 (pg/mL)
0.0033
0.0145
0.0035
0.0017
0.0050
Interleukin 17-A
IL17-A (pg/mL)
Interleukin 1-β
IL1-ß (pg/mL)
<0.0001
0.0057
0.0014
0.0017
0.0073
<0.0001
<0.0001
<0.0001
0.0148
0.0164
(A) Pro-Inflammatory Cytokines
IL-1β Stimulated
IL-1β + TNF Stimulated
LPS Stimulated
Growth-Regulated Oncogene
GRO (pg/mL)
0.0482
0.0345
0.0095
<0.0001
<0.0001
<0.0001
0.0009
0.0003
<0.0001
Tumor Necrosis Factor
TNF (pg/mL)
0.0003
0.0131
0.0025
0.0095
0.0115
Eotaxin
Eotaxin (pg/mL)
0.0079
(B) Anti-Inflammatory Cytokines
IL-1β Stimulated
IL-1β + TNF Stimulated
LPS Stimulated
Interleukin 10
IL-10 (pg/mL)
0.0198
0.0056
0.0022
LRS
Stimulated Control
MSCs (Horse 1)
MSCs (Horse 2)
MSCs (Horse 3)
ACPs (Horse 1)
ACPs (Horse 2)
ACPs (Horse 3)
