# Supplementary material for: Clonal articular cartilage progenitors outperform mesenchymal stromal cells in mitigating post-traumatic osteoarthritis
Source: Osteoarthr Cartil Open. 2026 Sep 25;8(4):100878. doi: 10.1016/j.ocarto.2026.100878 (PMC13683781; doi:10.1016/j.ocarto.2026.100878)
Supplement: Supplementary file 2 [file mmc4.pdf]

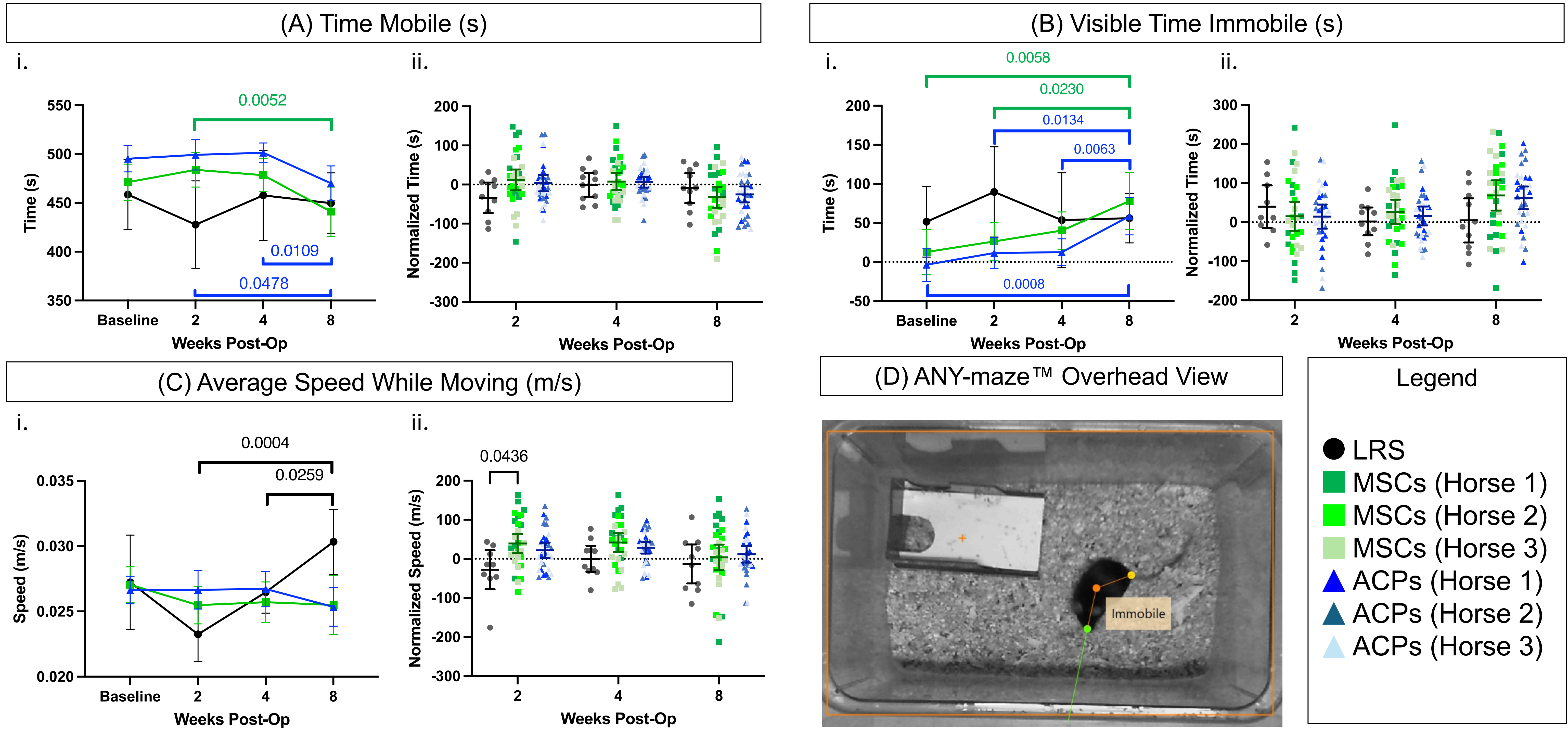
(A) Time Mobile (s)
i.
ii.
Time (s)
Normalized Time (s)
0.0052
0.0109
0.0478
Baseline
2
4
8
Weeks Post-Op
(B) Visible Time Immobile (s)
0.0058
0.0230
0.0134
0.0063
0.0008
(C) Average Speed While Moving (m/s)
Speed (m/s)
Normalized Speed (m/s)
0.0004
0.0259
0.0436
(D) ANY-maze™ Overhead View
Immobile
Legend
LRS
MSCs (Horse 1)
MSCs (Horse 2)
MSCs (Horse 3)
ACPs (Horse 1)
ACPs (Horse 2)
ACPs (Horse 3)
