# Supplementary material for: Clonal articular cartilage progenitors outperform mesenchymal stromal cells in mitigating post-traumatic osteoarthritis
Source: Osteoarthr Cartil Open. 2026 Sep 25;8(4):100878. doi: 10.1016/j.ocarto.2026.100878 (PMC13683781; doi:10.1016/j.ocarto.2026.100878)
Supplement: Supplementary file 3 [file mmc5.pdf]

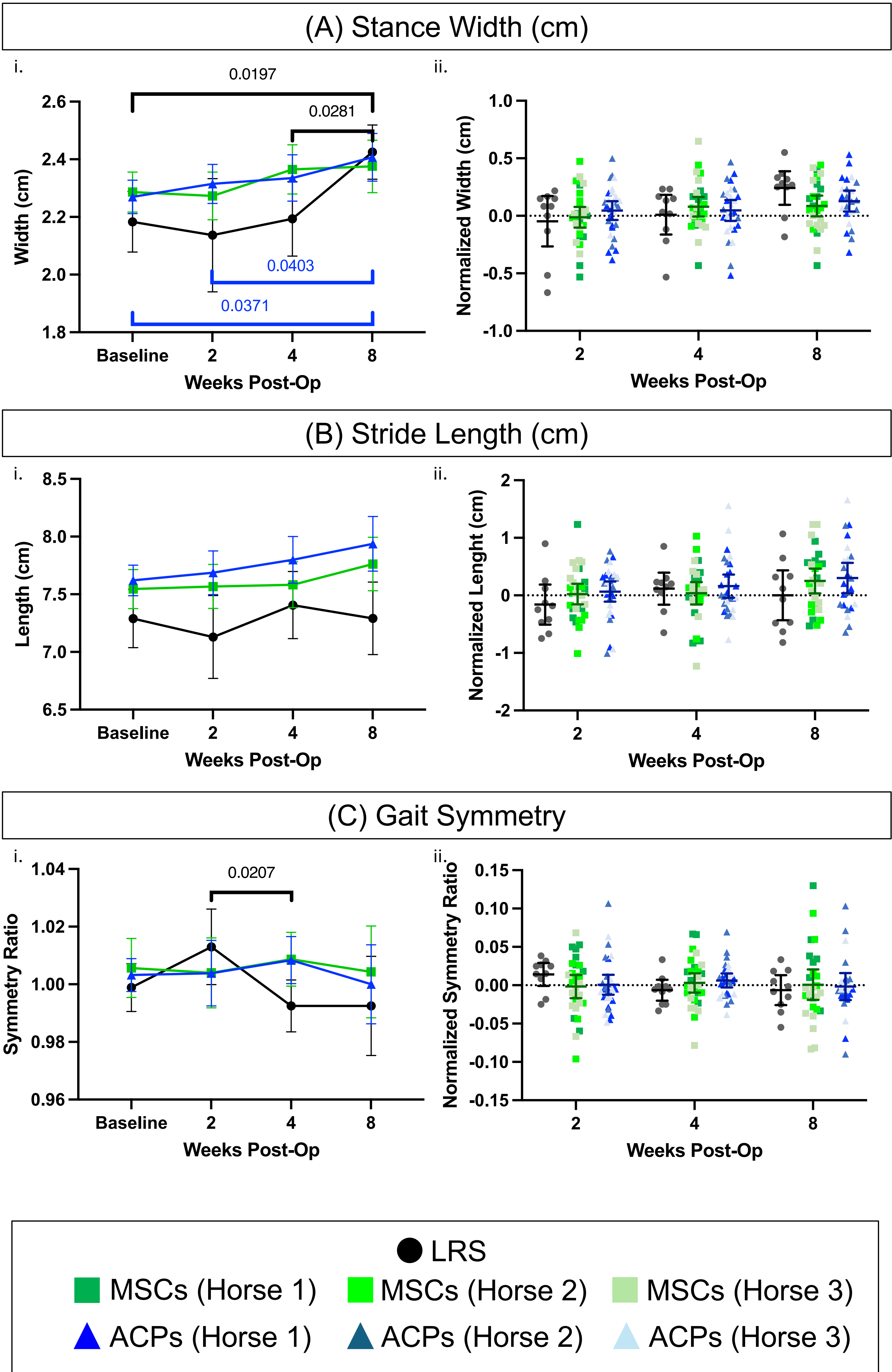

(A) Stance Width (cm)
i.
0.0197
0.0281
0.0403
0.0371
Width (cm)
Baseline
2
4
8
Weeks Post-Op
ii.
Normalized Width (cm)
Weeks Post-Op
(B) Stride Length (cm)
i.
Length (cm)
Baseline
Weeks Post-Op
ii.
Normalized Lenght (cm)
Weeks Post-Op
(C) Gait Symmetry
i.
0.0207
Symmetry Ratio
Baseline
Weeks Post-Op
ii.
Normalized Symmetry Ratio
Weeks Post-Op
LRS
MSCs (Horse 1)
MSCs (Horse 2)
MSCs (Horse 3)
ACPs (Horse 1)
ACPs (Horse 2)
ACPs (Horse 3)
