# Supplementary material for: Clonal articular cartilage progenitors outperform mesenchymal stromal cells in mitigating post-traumatic osteoarthritis
Source: Osteoarthr Cartil Open. 2026 Sep 25;8(4):100878. doi: 10.1016/j.ocarto.2026.100878 (PMC13683781; doi:10.1016/j.ocarto.2026.100878)
Supplement: Supplementary file 4 [file mmc6.pdf]

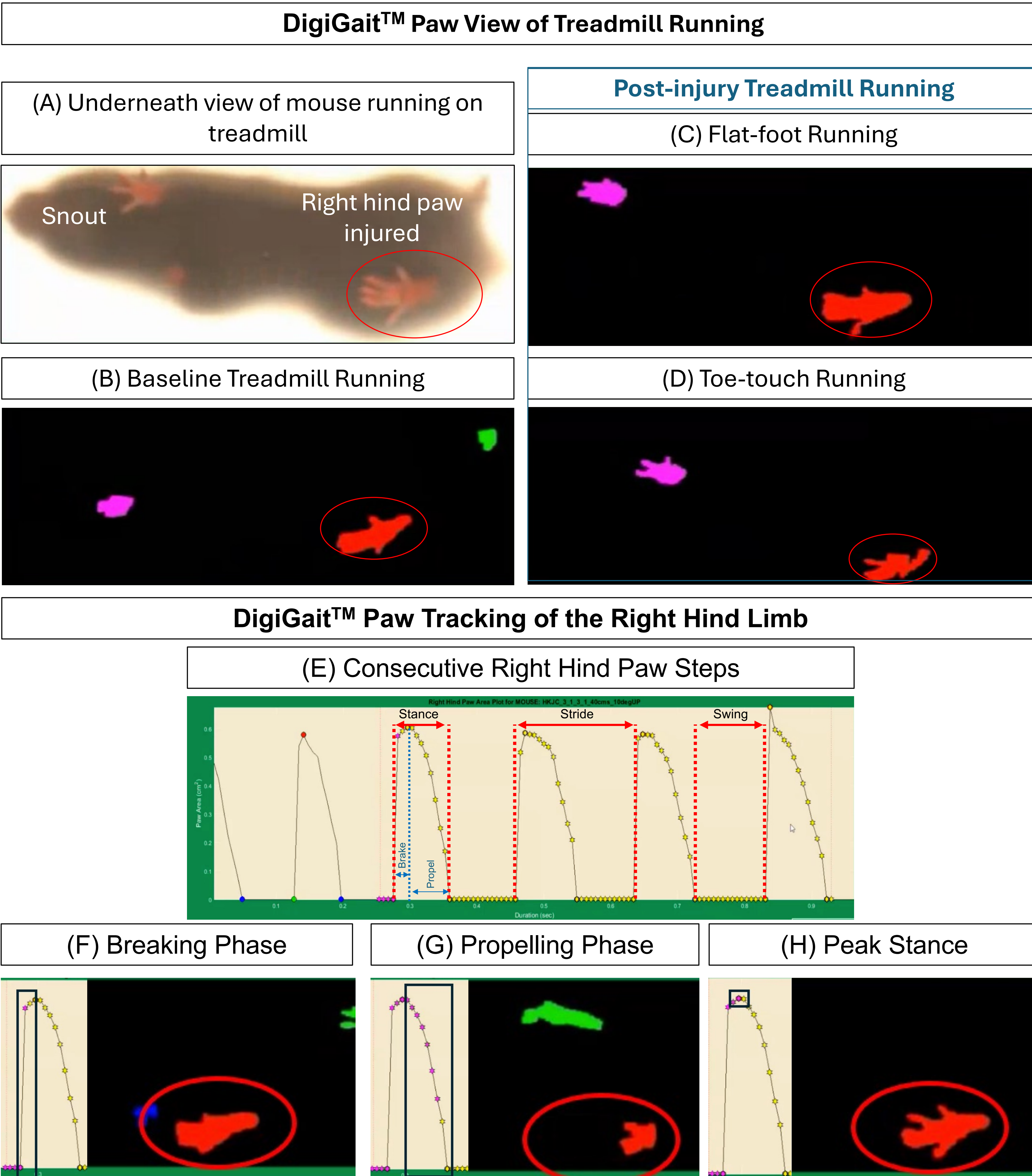
DigiGait™ Paw View of Treadmill Running
(A) Underneath view of mouse running on treadmill
Snout
Right hind paw injured
(B) Baseline Treadmill Running
Post-injury Treadmill Running
(C) Flat-foot Running
(D) Toe-touch Running
DigiGait™ Paw Tracking of the Right Hind Limb
(E) Consecutive Right Hind Paw Steps
Right Hind Paw Area Plot for MOUSE: HKJC_3_1_3_1_40cms_10degUP
Stance
Stride
Swing
Brake
Propel
Paw Area (cm²)
Duration (sec)
(F) Breaking Phase
(G) Propelling Phase
(H) Peak Stance
