# Supplementary material for: Clonal articular cartilage progenitors outperform mesenchymal stromal cells in mitigating post-traumatic osteoarthritis
Source: Osteoarthr Cartil Open. 2026 Sep 25;8(4):100878. doi: 10.1016/j.ocarto.2026.100878 (PMC13683781; doi:10.1016/j.ocarto.2026.100878)
Supplement: Supplementary file 5 [file mmc7.pdf]

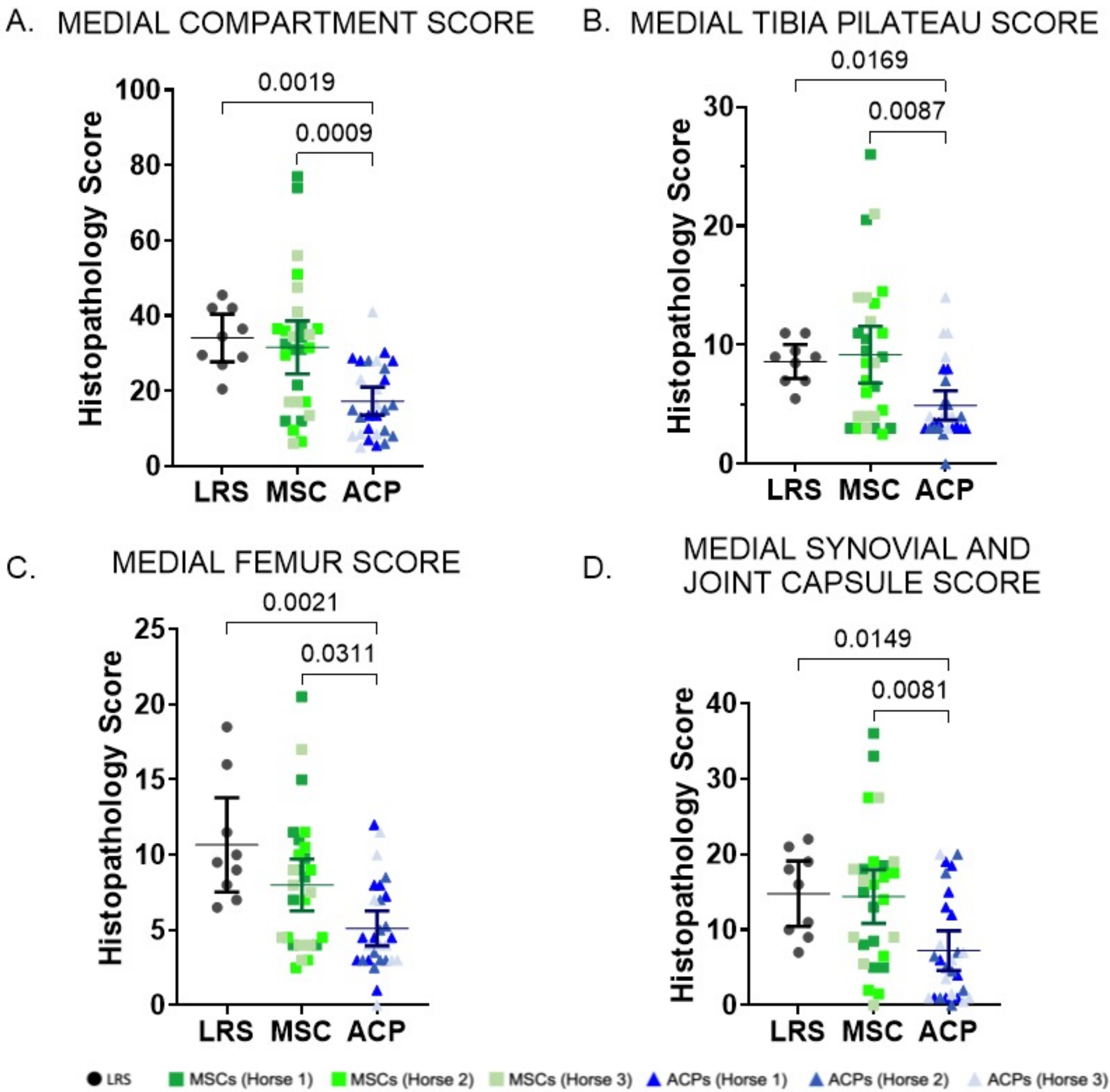

E.

Scoring Location	Treatment	Mean	Lower 95% CI	Upper 95% CI
Medial Compartment Scores	LRS	34.06	27.72	40.39
	MSC	31.56	24.52	38.61
	ACP	17.26	13.53	20.99
Medial Tibia Plateau Scores	LRS	8.611	7.189	10.03
	MSC	9.179	6.793	11.56
	ACP	4.92	3.692	6.147
Medial Femur Scores	LRS	10.67	7.533	13.8
	MSC	7.991	6.264	9.718
	ACP	5.107	3.95	6.265
Medial Synovial & Joint Compartment Scores	LRS	14.78	10.47	19.09
	MSC	14.39	10.85	17.94
	ACP	7.232	4.599	9.865
