# Supplementary material for: Clonal articular cartilage progenitors outperform mesenchymal stromal cells in mitigating post-traumatic osteoarthritis
Source: Osteoarthr Cartil Open. 2026 Sep 25;8(4):100878. doi: 10.1016/j.ocarto.2026.100878 (PMC13683781; doi:10.1016/j.ocarto.2026.100878)
Supplement: Supplementary file 6 [file mmc8.pdf]

Domain	Category	Score	Description
Domain 1: Cartilage (Medial Tibia and Femur only)	Cartilage Structure	0	normal/healthy
		1	<10% of cartilage depth: superficial fibrillation and/or rough surface
		2	>10% but not reaching not reaching calcified cartilage: loss of surface laminate and/or fibrillation depth
		3	Clefts to calcified cartilage with no loss of non-calcified cartilage or horizontal cracks between calcified and non-calcified cartilage
		4	<25% of joint surface: loss of non-calcified cartilage or fibrillation to calcified layer
		5	25-50% of joint surface: loss of non-calcified cartilage or fibrillation to calcified layer
		6	50-75% of joint surface: loss of non-calcified cartilage or fibrillation to calcified layer
		7	>75% of joint surface: loss of non-calcified cartilage or fibrillation to calcified layer
		8	<25% joint surface: lesion extending through calcified cartilage
		9	25-50% joint surface: lesion extending through calcified cartilage
		10	50-75% joint surface: lesion extending through calcified cartilage
		11	>75% joint surface: lesion extending through calcified cartilage
	Cartilage Proteoglycan	0	normal/healthy
		1	Decreased toluidine blue staining
		2	<25% of joint surface: loss of toluidine blue staining
		3	25-50% of joint surface: loss of toluidine blue staining
		4	50-75% of joint surface: loss of toluidine blue staining
		5	>75% of joint surface: loss of toluidine blue staining
	Cartilage Cellularity	0	no hypertrophy/apoptosis
		1	Chondrocyte chondrones
		2	Chondrocyte clustering
		3	<33% of cells missing
		4	33 - 66% of cells missing
		5	>66% of cells missing
Domain 4: Synovium (Medial compartment only)	Pannus	0	normal/healthy (no panus)
		1	>2 cell thickness pannus present at bone and joint margin but not migrated over cartilage surface
		2	<1 x cartilage depth, panus has migrated over cartilage surface OR invaded cartilage
		3	>1 x cartilage depth, panus has migrated over cartilage surface OR invaded cartilage
	Bone Erosion	0	normal/healthy (no cortical bone erosion at any site)
		1	partial thickness loss of cortical bone only
		2	focal complete loss of cortical bone in one small vascular site
		3	widespread complete loss of cortical bone multiple vascular sites or broad area loss of cortical bone
	Synovial Hyperplasia	0	normal/healthy
		1	1-2 cell layers thick
		2	3-4 cell layers thick
		3	5+ cell layers thick
	Inflammation/ Infiltration	0	normal/healthy (no inflammatory cells)
		1	occasional scattered inflammatory cells
		2	FOCAL areas of dense sub-synovial white blood cell infiltrate, normal connective tissue present
		3	WIDESPREAD dense sub-synovial white blood cell infiltrate, reduced areolar connective tissue, OR LYMPHOID FOLLICLE formation
	Exudate	0	normal/healthy
		1	inflammatory cells and/or fibrin clot in the synovial cavity
	Synovial/Joint-Capsule Fibrosis	A	Fibrosis Score -score the thickest bit of fibrosis irrespective of the % of the synovium
		0	<33% depth of cartilage
		1	33 - 66% depth of cartilage, NOT densely packed fibrous tissue
		2	33 - 66% depth of cartilage, densely packed fibrous tissue
		3	66% - 1x depth of cartilage, NOT densely packed fibrous tissue
		4	66% - 1x depth of cartilage, densely packed fibrous tissue
		5	>1x depth of cartilage, NOT densely packed fibrous tissue
		6	>1x depth of cartilage, densely packed fibrous tissue
		B	Fibrosis Spread - score how much each depth/severity is present across the total synovium region being evaluate
		0	<20%
		1	21 - 40%
		2	41 - 60%
		3	61 - 80%
		4	>80%
		C	Fibrosis severity score = score*spread
Domain 2: Bone (Osteophyte Formation/Maturation; Medial Tibia and Femur only)	Osteocyte Death Subchondral Bone (only Tibia)	0	normal/healthy
		0.5	occasional empty lacunae, scattered
		1	<33% osteophytes affected
		2	33 - 66% osteocytes affected
		3	>66% osteocytes affected
	Sclerosis Subchondral Bone (only Tibia)	0	normal/healthy (max 2 thin bone struts <50% of tibial cartilage depth)
		1	2 or more wide bone struts spanning >50% depth of tibial cartilage, and vertical trabecular struts extending from cartilage to growth plate/anterior patella, OR solid bone spanning up to 33% of epiphysis/patella width
		2	solid bone spanning 33 - 66% epiphysis/patella width
		3	solid bone spanning >66% epiphysis/patella width
	Vascular Erosion/invasion Subchondral Bone (only Tibia)	0	normal/healthy (intact subchondral bone layer across entire surface)
		1	1 - 2 points of vascular invasion that touch, but not invade articular cartage
		2	≥3 points of vascular invasion that touch, but not invade articular cartage, OR any number of points that invade into calcified articular cartilage but do not cross tidemark
		3	any number of points that invade calcified articular cartilage and cross into the tidemark (including pockets of inflammatory/fibrovascular/synovial cells present in the non-calcified articular cartilage lacking channels to the subchondral bone)
	Osteochondral Damage (only Tibia)	0	normal/healthy
		1	cracks near joint margin and close/within growth plate/anterior patella
		2	cracks in bone at osteochondral junction & no overlying articular cartilage pathology
		3	Cracks through osteochondral junction into articular cartilage & no overlying articular cartilage pathology
		4	Cracks through osteochondral junction into articular cartilage + surface defects in the overlying articular cartilage
		5	<33% depth of epiphysis/patella erosion of subchondral bone
		6	33 - 66% of depth of epiphysis/patella erosion of subchondral bone
		7	>66% of depth of epiphysis/patella erosion of subchondral bone not eroding the growth plate/anterior patella
		8	<33% of joint, erosion into/loss of growth plate
		9	33 - 66% of joint, erosion into/loss of growth plate
		10	>66% of joint, erosion into/loss of growth plate
	Osteophyte Formation/maturation	A	Maturity
		0	normal/healthy (no osteophyte/remodeling)
		0.5	Predominantly fibrous tissue – no chondroid metaplasia
		1	Predominantly cartilaginous - no active endochondral ossification; may have fibrous tissue with chondroid metaplasia
		2	Mixed cartilage and bone with active endochondral ossification – chondrocyte hypertrophy and active vascular invasion and new bone formation
		3	Predominantly bone with NO active endochondral ossification (can have chondrocyte like cells or cartilage cap present over bone but NO active vascular invasion)
		B	Size
		0	normal/healthy
		1	<1x (same) thickness of adjacent normal cartilage
		2	1 - 3x thickness of adjacent normal cartilage
		3	3 - 5x thickness of adjacent normal cartilage
		4	> 5x thickness of adjacent normal cartilage
		C	Osteophyte severity score = Maturity*Size
	Enthesophyte Formation/Maturation	A	Maturity
		0	normal/healthy (no osteophyte/remodeling)
		0.5	Predominantly fibrous tissue – no chondroid metaplasia
		1	Predominantly cartilaginous - no active endochondral ossification; may have fibrous tissue with chondroid metaplasia
		2	Mixed cartilage and bone with active endochondral ossification – chondrocyte hypertrophy and active vascular invasion and new bone formation
		3	Predominantly bone with NO active endochondral ossification (can have chondrocyte like cells or cartilage cap present over bone but NO active vascular invasion)
		B	Size
		0	normal/healthy
		1	<1x (same) thickness of adjacent normal cartilage
		2	1 - 3x thickness of adjacent normal cartilage
		3	3 - 5x thickness of adjacent normal cartilage
		4	> 5x thickness of adjacent normal cartilage
		C	Enthesophyte severity score = Maturity*Size
