# Supplementary material for: Clonal articular cartilage progenitors outperform mesenchymal stromal cells in mitigating post-traumatic osteoarthritis
Source: Osteoarthr Cartil Open. 2026 Sep 25;8(4):100878. doi: 10.1016/j.ocarto.2026.100878 (PMC13683781; doi:10.1016/j.ocarto.2026.100878)
Supplement: Supplementary file 7 [file mmc9.pdf]

Figure	Type	Paramater	Comparison	p-value	95% CI of diff.	Variable 1 (V1)	V1 mean	V1 95% CI lower	V1 95% CI upper	Variable 2 (v2)	V2 mean	V2 95% CI lower	V2 95% CI upper
Figure 2 A	IL-1ß stimulated	Il-1ß	Control vs. IL1-β	<0.0001	[-1865, -831.5]	control	0	0	0	IL-1ß	1348.0	1150.0	1547.0
	IL-1ß stimulated	Il-1ß	Control vs. MSC + IL1-β	0.0017	[-1171, -275.9]	control	0	0	0	MSC+IL-1ß	723.5	369.9	1077.0
	IL-1ß stimulated	Il-1ß	Control vs. ACP + IL1-β	0.0073	[-1053, -157.7]	control	0	0	0	ACP+IL-1ß	605.3	469.7	741.0
	IL-1ß stimulated	Il-1ß	IL1-β vs. MSC + IL1-β	0.0057	[177.2, 1072]	IL-1ß	1348	1150	1547	MSC+IL-1ß	723.5	369.9	1077.0
	IL-1ß stimulated	Il-1ß	IL1-β vs. ACP + IL1-β	0.0014	[295.4, 1191]	IL-1ß	1348	1150	1547	ACP+IL-1ß	605.3	469.7	741.0
Figure 2 B	IL-1ß + TNF stimulated	Il-1ß	Control vs. IL1-β+TNF	<0.0001	[-2386, -1457]	control	0	0	0	IL1-β+TNF	1922.0	1548.0	2295.0
	IL-1ß + TNF stimulated	Il-1ß	Control vs. MSC + IL1-β+TNF	0.0148	[-894.7, -89.77]	control	0	0	0	MSC + IL1-β+TNF	492.2	326.4	658.1
	IL-1ß + TNF stimulated	Il-1ß	Control vs. ACP + IL1-β+TNF	0.0164	[-887.6, -82.68]	control	0	0	0	ACP + IL1-β+TNF	485.1	201.0	769.3
	IL-1ß + TNF stimulated	Il-1ß	IL1-β+TNF vs. MSC + IL1-β+TNF	<0.0001	[1027, 1832]	IL1-β+TNF	1922	1548	2295	MSC + IL1-β+TNF	492.2	326.4	658.1
	IL-1ß + TNF stimulated	Il-1ß	IL1-β+TNF vs. ACP + IL1-β+TNF	<0.0001	[1034, 1839]	IL1-β+TNF	1922	1548	2295	ACP + IL1-β+TNF	485.1	201.0	769.3
Figure 2 C	IL-1ß stimulated	IP-10	Control vs. MSC + IL1-β	0.0285	[4.39, 88.83]	control	94.41	16.28	172.5	MSC + IL1-β	47.8	25.4	70.2
	IL-1ß stimulated	IP-10	MSC + IL1-β vs. ACP + IL1-β	0.0302	[-72.16, -3.22]	MSC + IL1-β	47.8	25.38	70.21	ACP + IL1-β	85.5	66.5	104.5
Figure 2 D	IL-1ß + TNF stimulated	Eotaxin	IL1-β+TNF vs. ACP + IL1-β+TNF	0.0079	[0.64, 4.41]	IL1-β+TNF	19.35	19.35	19.35	ACP + IL1-β+TNF	16.8	15.8	17.9
Figure 2 E	IL-1ß stimulated	IL-10	Control vs. ACP + IL1-β	0.0351	[-1426, -45.47]	control	1036	289.8	1781	ACP+IL-1ß	1772.0	1232.0	2311.0
	IL-1ß stimulated	IL-10	MSC + IL1-β vs. ACP + IL1-β	0.0005	[-1601, -473.2]	MSC + IL1-β	734.6	612.7	856.4	ACP + IL1-β	1772.0	1232.0	2311.0
Figure 2 F	IL-1ß + TNF stimulated	IL-10	Control vs. ACP + IL1-β+TNF	0.0022	[-2006, -444.3]	control	1036	289.8	1781	ACP + IL1-β+TNF	2261.0	1733.0	2788.0
	IL-1ß + TNF stimulated	IL-10	MSC + IL1-β+TNF vs. ACP + IL1-β+TNF	0.0056	[-1530, -255]	MSC + IL1-β+TNF	1368	1017	1720	ACP + IL1-β+TNF	2261.0	1733.0	2788.0
Supp. Fig. 1 IL-1ß Stimulated	IL-1ß stimulated	IP-10	See Figure 2 C above										
	IL-1ß stimulated	Il-1ß	See Figure 2 A above										
	IL-1ß stimulated	IL-10	See Figure 2 E above										
Supp. Fig. 1 IL-1ß Stimulated	IL-1ß stimulated	GRO	Control vs. IL1-β	0.0482	[-293.2, -1]	control	234.5	157.7	311.3	IL-1ß	381.6	378.6	384.7
	IL-1ß stimulated	GRO	Control vs. MSC + IL1-β	0.0345	[-261.8, -8.72]	control	234.5	157.7	311.3	MSC+IL-1ß	369.8	278.6	460.9
	IL-1ß stimulated	GRO	Control vs. ACP + IL1-β	0.0095	[-291.6, -38.53]	control	234.5	157.7	311.3	ACP + IL1-β	399.6	345.2	454.0
Supp. Fig. 1 IL-1ß+TNF Stimulated	IL-1ß + TNF stimulated	Il-1ß	See Figure 2 B above										
	IL-1ß + TNF stimulated	Eotaxin	See Figure 2 D above										
	IL-1ß + TNF stimulated	IL-10	See Figure 2 F above										
Supp. Fig. 1 IL-1ß+TNF Stimulated	IL-1ß + TNF stimulated	IP-10	Control vs. IL1-β+TNF	0.0486	[0.23, 89.46]	control	94.41	16.28	172.5	IL1-β+TNF	49.6	42.5	56.7
	IL-1ß + TNF stimulated	IP-10	Control vs. MSC + IL1-β+TNF	0.0026	[20.77, 98.04]	control	94.41	16.28	172.5	MSC + IL1-β+TNF	35.0	19.9	50.1
	IL-1ß + TNF stimulated	IP-10	Control vs. ACP + IL1-β+TNF	0.0006	[31.95, 109.2]	control	94.41	16.28	172.5	ACP + IL1-β+TNF	23.8	3.3	44.4
Supp. Fig. 1 IL-1ß+TNF Stimulated	IL-1ß + TNF stimulated	IL-8	Control vs. IL1-β+TNF	0.0033	[-472.1, -93.97]	control	76.71	59.02	94.4	IL1-β+TNF	359.7	323.1	396.4
	IL-1ß + TNF stimulated	IL-8	Control vs. MSC + IL1-β+TNF	0.0145	[-364.7, -37.25]	control	76.71	59.02	94.4	MSC + IL1-β+TNF	277.7	159.1	396.3
	IL-1ß + TNF stimulated	IL-8	Control vs. ACP + IL1-β+TNF	0.0035	[-407.1, -79.67]	control	76.71	59.02	94.4	ACP + IL1-β+TNF	320.1	246.8	393.5
Supp. Fig. 1 IL-1ß+TNF Stimulated	IL-1ß + TNF stimulated	GRO	Control vs. IL1-β+TNF	<0.0001	[-261.5, -152.7]	control	234.5	157.7	311.3	IL1-β+TNF	441.6	427.4	455.8
	IL-1ß + TNF stimulated	GRO	Control vs. MSC + IL1-β+TNF	<0.0001	[-242.8, -148.6]	control	234.5	157.7	311.3	MSC + IL1-β+TNF	430.2	400.7	459.7
	IL-1ß + TNF stimulated	GRO	Control vs. ACP + IL1-β+TNF	<0.0001	[-266.8, -172.6]	control	234.5	157.7	311.3	ACP + IL1-β+TNF	454.2	436.4	472.0
Supp. Fig. 1 IL-1ß+TNF Stimulated	IL-1ß + TNF stimulated	TNF	Control vs. IL1-β+TNF	0.0003	[-366, -116.5]	control	31.4	-7.775	70.57	IL1-β+TNF	272.7	232.6	312.7
	IL-1ß + TNF stimulated	TNF	Control vs. MSC + IL1-β+TNF	0.0131	[-242.7, -26.57]	control	31.4	-7.775	70.57	MSC + IL1-β+TNF	166.0	97.2	234.8
	IL-1ß + TNF stimulated	TNF	Control vs. ACP + IL1-β+TNF	0.0025	[-275.1, -59.05]	control	31.4	-7.775	70.57	ACP + IL1-β+TNF	198.0	138.8	258.2
Supp. Fig. 1 LPS Stimulated	LPS stimulated	IP-10	Control vs. LPS	<0.0001	[57.72, 131.1]	control	94.41	16.28	172.5	LPS	0.0	0.0	0.0
	LPS stimulated	IP-10	Control vs. LPS + MSC	0.0034	[15.6, 79.15]	control	94.41	16.28	172.5	LPS + MSC	47.0	43.0	51.1
	LPS stimulated	IP-10	Control vs. LPS + ACP	<0.0001	[37.43, 101]	control	94.41	16.28	172.5	LPS + ACP	25.2	8.3	42.1
	LPS stimulated	IP-10	LPS vs. LPS + MSC	0.0036	[-78.8, -15.25]	LPS	0	0	0	LPS + MSC	47.0	43.0	51.1
Supp. Fig. 1 LPS Stimulated	LPS stimulated	IL-8	Control vs. LPS + MSC	0.0017	[-210.3, -49.49]	control	76.71	59.02	94.4	LPS + MSC	206.6	177.9	235.3
	LPS stimulated	IL-8	Control vs. LPS + ACP	0.005	[-194.6, -33.78]	control	76.71	59.02	94.4	LPS + ACP	190.9	164.9	216.9
Supp. Fig. 1 LPS Stimulated	LPS stimulated	GRO	Control vs. LPS	0.0009	[-212.8, -57.28]	control	234.5	157.7	311.3	LPS	369.6	225.4	513.8
	LPS stimulated	GRO	Control vs. LPS + MSC	0.0003	[-200.1, -65.44]	control	234.5	157.7	311.3	LPS + MSC	367.3	335.1	399.5
	LPS stimulated	GRO	Control vs. LPS + ACP	<0.0001	[-247.2, -112.5]	control	234.5	157.7	311.3	LPS + ACP	414.4	395.3	433.6
Supp. Fig. 1 LPS Stimulated	LPS stimulated	TNF	Control vs. LPS	0.0095	[-66.4, -8.75]	control	31.4	-7.78	-7.78	LPS	69.0	50.2	87.8
	LPS stimulated	TNF	LPS vs. LPS + MSC	0.0115	[6.73, 56.65]	LPS	68.97	50.19	87.76	LPS + MSC	37.3	25.8	48.8
