# Supplementary material for: Clonal articular cartilage progenitors outperform mesenchymal stromal cells in mitigating post-traumatic osteoarthritis
Source: Osteoarthr Cartil Open. 2026 Sep 25;8(4):100878. doi: 10.1016/j.ocarto.2026.100878 (PMC13683781; doi:10.1016/j.ocarto.2026.100878)
Supplement: Supplementary file 8 [file mmc1.docx]

**Supplemental:**

**Methods:**

Monocyte-derived macrophage cell culture.

Equine peripheral blood mononuclear cells were isolated from whole blood from one donor horse (three-year-old Quarter Horse gelding) by density gradient centrifugation (Ficoll-Paque TM+, CYTIVA, Product:# 17144003). Cells were plated at 5x10^6^ cells/well on 24-well plates and for 18 hours in complete growth media, then washed thrice in phosphate-buffered saline (PBS) and cultured in macrophage media (DMEM with 10% FBS, non-essential amino acids, and penicillin/streptomycin; DMEM: Corning 10-014-CV, FBS: Atlas Biologicals F-0500-DR, Non ess AA: Gibco 11140-050, Pen/strep: Gibco 15140-122)) with human macrophage colony-stimulating factor (M-CSF) (PeproTech at 30 ng/mL) (Peprotech 300-25) to stimulate differentiation into macrophages for five days^1^ (**Fig. 1**).

Macrophage Stimulation.

On day six in culture, media was removed from equine monocyte-derived macrophages and replaced with a 1:1 stimulation media (macrophage media: conditioned from ACPs or MSCs) treated with stimulatory cytokine(s) interleukin-1ß (IL-1ß) (10ng/mL, Peprotech 200-01B) or IL-1ß (10ng/mL) and tumor necrosis factor (TNF) (10µg/mL, Peprotech 300-01A) for 48 hours; these cytokines are highly associated with cartilage degeneration and shown to activate synovial macrophages^2,3^. Controls included unstimulated macrophages in macrophage growth media or macrophages stimulated with IL-1ß or IL-1ß + TNF. After stimulation, macrophages were washed thrice with PBS and cultured for 24 hours in macrophage growth media. Supernatants were collected, aliquoted, and frozen at -80°C for later use in the multiplex bead assay to characterize the macrophage cytokine response (**Fig. 1**).

Surgical induction of PTOA.

PTOA was induced in the right hind stifle (knee) using the well-established DMM method^4^. Briefly, mice were anesthetized, administered 1 mg/kg slow-release buprenorphine (a non-anti-inflammatory pain killer lasting 48-72hrs)^5,6^, and placed supine for aseptic preparation of the surgical site. An approximately 3mm longitudinal incision was made using a #15 blade overlying the distal patella of the right hind limb to the proximal tibia plateau^4,7^. The infrapatellar fat pad (IFP) was laterally repositioned temporarily to allow access to the anterior medial meniscotibial ligament. The anchoring point of the medial meniscotibial ligament to the tibial plateau was severed using a #11 scalpel blade. Following destabilization, the IFP was repositioned, and the arthrotomy closed using a 6-0 monofilament absorbable suture in a simple interrupted pattern. Contralateral limbs served as unoperated naïve controls^4,7^.

Exclusion.

Total animals included in the ANY-maze analysis at baseline (if one baseline did not capture the other, the other baseline was used for these animals instead of averaged, if an animal did at least one baseline, it is reported here), LRS n=10, ACP n=30, MSC n=30. Week 2 LRS n=9, ACP n=30, MSC n=29. Week 4 LRS n=10, ACP n=30, MSC n=29. Week 8 LRS n=10, ACP n=, MSC n=29.

Total animals included in the DigiGait analysis at baseline (if one baseline did not capture the other, the other baseline was used for these animals instead of averaged, if an animal did at least one baseline, it is reported here), LRS n=10, ACP n=29, MSC n=28. Week 2 LRS n=10, ACP n=29, MSC n=28. Week 4 LRS n=10, ACP n=27, MSC n=28. Week 8 LRS n=10, ACP n=23, MSC n=26.

Animals were excluded from histology if sectioning was ungradable. Animals were deemed ungradable after two embedding and sectioning attempts, for reasons such as shearing during sectioning. Total gradable animals by groups LRS n=9, ACP n=24, MSC n=23.

Statistical Analysis.

The experimental sample size and power calculations for the in vivo study were determined using the statistical software developed by R.V. Lenth, which is available at: <http://www.stat.uiowa.edu/~rlenth/Power>. Based on pilot studies and previous work performed by the principal investigator, histologic assessment of OA was chosen as the primary outcome measure, with secondary outcomes of behavior and mobility. A two-way ANOVA was used to estimate the within-group variance as 1.0 and a detectable contrast as 1.0. Using these values, power associated with a Tukey/HSD post-test (alpha = 0.05) was calculated as 0.9674 with a sample size of 10 per group. Secondary outcomes from previously published work also support meaningful statistical differences with group sizes of 10^7^.

To account for potential donor variability, horse (cell donor) was included as a variable in the statistical models. No significant donor-dependent effects were observed across outcome measures, supporting the pooling of animals within treatment groups for analysis. Mouse weights were evaluated individually over time to verify no animals needed to be excluded for other health concerns, and longitudinally by treatment group with a mixed linear regression model.

**Supplementary Tables 5–8** provide the complete statistical output corresponding to the summarized results presented in **Table 3.**

**Figures/Tables:**

**Supplementary Table 1: Histology Scoring Parameters and Scores**

**Supplementary Table 2: ANY-maze Tests Excluded from Analysis**

| Cohort | CCA_ID | Treatment | Week | Error | Reason/Notes |
| --- | --- | --- | --- | --- | --- |
| 3 | 31.3 | Control | 0.2 | All | likely computer glitch/tracking errors |
| 3 | 31.1 | Control | 2 | Time freezing | Time freezing was very far outside of all other measures, concluded the computer is likely glitching. Removed all measures for now. |
| 4 | 41.2 | H1T1 | 0.1 | Top of Hut : time immobile | Very far from other measures and second baseline |
| 5 | 51.5 | H1T1 | 0.2 | Max speed | Not physiologically possible |
| 4 | 42.5 | H1T2 | 0.1 | Max speed | Not physiologically possible |
| 6 | 61.1 | H2T1 | 0.2 | All | likely computer glitch/tracking errors |
| 7 | 71.1 | H2T1 | 2 | All | likely computer glitch/tracking errors |
| 7 | 71.5 | H2T1 | 4 | All | likely computer glitch/tracking errors |
| 7 | 71.2 | H2T1 | 8 | All | likely computer glitch/tracking errors |
| 7 | 72.5 | H2T2 | 2 | All | likely computer glitch/tracking errors, probably nocked over hut |
| 7 | 72.2 | H2T2 | 0.2 | Max speed | Not physiologically possible |
| 8 | 81.1 | H3T1 | 0.1 | All | likely computer glitch/tracking errors |
| 8 | 82.2 | H3T2 | 0.2 | All | likely computer glitch/tracking errors |
| 9 | 92.3 | H3T2 | 2 | Max speed | Not physiologically possible |

**Supplementary Table 3: DigiGait Runs Excluded from Analysis**

| Cohort | ID | Treatment | Week | Paw | Error | Run | Reason/Notes |
| --- | --- | --- | --- | --- | --- | --- | --- |
| 3 | 1.2 | con | 4 | RH | Stride length | 3d | CV= 83.34 |
| 3 | 1.3 | con | 0.1 | RH | Stride length | 3f | CV=21.62 |
| 3 | 1.4 | con | 2 | RH | Stride length | 1f | Cv=14.21 |
| 3 | 1.4 | con | 4 | RH | Stride length | 2f | Cv=23.05 |
| 4 | 1.5 |  | 0.2 | RH | Stride length | 1f | cv=11.13 |
| 4 | 2.2 |  | 0.2 | RH | Stride length | 2i | cv= 12.03 |
| 4 | 2.3 |  | 4 | RH | Stride length | 1d | cv= 10.21 |
| 4 | 2.4 |  | 8 | RH | Stride length | 1d | cv=73.12 |
| 4 | 2.5 |  | 4 | RH | Stride length | 2f | cv= 10.2 |
| 4 | 2.5 |  | 8 | RH | Stride length | 3i | cv=122.03 |
| 5 | 1.1 |  | 0.2 | RH | Stride length | 2f | cv=54.12 |
| 5 | 1.1 |  | 0.2 | RH | Stride length | 3d | cv=31.55 |
| 5 | 1.3 |  | 8 | RH | Stride length | 2f | cv=12.83 |
| 5 | 1.3 |  | 4 | RH | Stride length | 3i | cv=29.62 |
| 5 | 2.2 |  | 2 | RH | Stride length | 1f | cv=27.85 |
| 5 | 2.2 |  | 8 | RH | Stride length | 1d | cv=42.41 |
| 5 | 2.4 |  | 4 | RH | Stride length | 1f | cv=30.16 |
| 6 | 1.1 |  | 4 | RH | Stride length | 2d | cv=25 |
| 6 | 1.3 |  | 0.1 | RH | Stride length | 2f | cv=19.65 |
| 6 | 1.3 |  | 0.1 | RH | Stride length | 1d | cv=14.52 |
| 6 | 1.3 |  | 4 | RH | Stride length | 3d | cv=14.07 |
| 6 | 1.4 |  | 8 | RH | Stride length | 1f | cv=23.31 |
| 6 | 2.1 |  | 0.2 | RH | Stride length | 1f | cv=23.81 |
| 6 | 2.1 |  | 4 | RH | Stride length | 1f | cv=15.49 |
| 6 | 2.1 |  | 0.1 | RH | Stride length | 2i | cv=14.85 |
| 6 | 2.1 |  | 0.2 | RH | Stride length | 1d | cv=15.04 |
| 6 | 2.2 |  | 0.1 | RH | Stride length | 3f | cv=40.46 |
| 6 | 2.2 |  | 0.1 | RH | Stride length | 3i | cv=13.82 |
| 6 | 2.3 |  | 4 | RH | Stride length | 1d | cv=77.39 |
| 6 | 2.5 |  | 8 | RH | Stride length | 3f | cv=22.87 |
| 6 | 2.5 |  | 0.1 | RH | Stride length | 3d | cv=104.08 |
| 7 | 1.1 |  | 4 | RH | Stride length | 1f | cv=27.13 |
| 7 | 1.3 |  | 8 | RH | Stride length | 2d | cv=18.07 |
| 7 | 1.4 |  | 2 | RH | Stride length | 2d | cv=23.88 |
| 7 | 1.5 |  | 0.1 | RH | Stride length | 3i | cv=28.85 |
| 7 | 2.1 |  | 2 | RH | Stride length | 1f | cv=44.77 |
| 7 | 2.1 |  | 2 | RH | Stride length | 1d | cv=25.39 |
| 7 | 2.2 |  | 4 | RH | Stride length | 2f | cv=30.24 |
| 7 | 2.2 |  | 8 | RH | Stride length | 1f | cv=14.36 |
| 7 | 2.2 |  | 0.2 | RH | Stride length | 3d | cv=34.5 |
| 7 | 2.3 |  | 0.1 | RH | Stride length | 1i | cv=124.32 |
| 7 | 2.4 |  | 2 | RH | Stride length | 2f | cv=31.77 |
| 7 | 2.5 |  | 0.2 | RH | Stride length | 2f | stride length= 22.3 NPP |
| 7 | 2.5 |  | 4 | RH | Stride length | 2i | cv=12.28 |
| 7 | 2.5 |  | 0.2 | RH | Stride length | 3d | cv=20.68 |
| 8 | 1.1 |  | 0.2 | RH | Stride length | 3d | cv=17.91 |
| 8 | 1.1 |  | 8 | RH | Stride length | 3d | cv=31.22 |
| 8 | 1.3 |  | 0.2 | RH | Stride length | 3f | cv=16.35 |
| 8 | 1.3 |  | 8 | RH | Stride length | 3f | cv=39.66 |
| 8 | 2.1 |  | 8 | RH | Stride length | 1f | cv=17.8 |
| 8 | 2.4 |  | 4 | RH | Stride length | 2i | cv=17.91 |
| 3 | 1.2 |  | 4 | RH | Stride length | 3i | Stride length= 11.4 NPP |
| 3 | 1.5 |  | 4 | RH | Stride length | 1f | SL=14.2 |
| 3 | 2.4 |  | 4 | RH | Stride length | 1d | SL=11.8 |
| 4 | 1.2 |  | 8 | RH | Stride length | 3f | sl=11.6 |
| 4 | 1.4 |  | 0.1 | RH | Stride length | 1f | sl=17.9 |
| 4 | 1.4 |  | 0.1 | RH | Stride length | 1i, 2i, 3i | sl=17.9, 20.2, 18.7 |
| 4 | 1.4 |  | 0.1 | RH | Stride length | 2d, 3d | sl=18.4, 18.2 |
| 4 | 1.4 |  | 8 | RH | Stride length | 1d | sl=17.3 |
| 4 | 1.4 |  | 8 | RH | Stride length | 3d | sl=21.1 |
| 4 | 2.2 |  | 0.2 | RH | Stride length | 1f | sl=14.6 |
| 4 | 2.2 |  | 8 | RH | Stride length | 2f | sl=19 |
| 4 | 2.2 |  | 8 | RH | Stride length | 3i | sl=11.6 |
| 4 | 2.5 |  | 8 | RH | Stride length | 1f | sl=16.7 |
| 4 | 2.5 |  | 8 | RH | Stride length | 3d | sl=11.2 |
| 5 | 1.3 |  | 0.2 | RH | Stride length | 1f | sl=11.5 |
| 5 | 1.4 |  | 2 | RH | Stride length | 3i | sl=11.4 |
| 5 | 1.5 |  | 2 | RH | Stride length | 2d | sl=11.6 |
| 5 | 2.5 |  | 2 | RH | Stride length | 1i | sl=11 |
| 6 | 1.5 |  | 8 | RH | Stride length | 1d | sl=18.8 |
| 6 | 2.1 |  | 4 | RH | Stride length | 1d | sl=11.8 |
| 6 | 2.3 |  | 8 | RH | Stride length | 3i | sl=13.5 |
| 6 | 2.5 |  | 0.1 | RH | Stride length | 1i | sl=13.4 |
| 7 | 2.1 |  | 4 | RH | Stride length | 3d | sl=13.7 |
| 9 | 1.2 |  | 8 |  | Stride length | 1f | cv= 25.11 |
| 9 | 1.5 |  | 0.1 |  | Stride length | 2f | cv=24.37 |
| 9 | 2.2 |  | 0.2 |  | Stride length | 2d | cv= 32.53 |
| 9 | 2.5 |  | 4 |  | Stride length | 3f | cv=17.51 |

References:

```
1.	Ma J, Wang SS, Lin YZ, et al. Infection of equine monocyte-derived macrophages with an attenuated equine infectious anemia virus (EIAV) strain induces a strong resistance to the infection by a virulent EIAV strain. Vet Res. 2014;45(1):82. doi:10.1186/s13567-014-0082-y
2.	Caron JP, Gandy JC, Brown JL, Sordillo LM. Omega-3 fatty acids and docosahexaenoic acid oxymetabolites modulate the inflammatory response of equine recombinant interleukin1β-stimulated equine synoviocytes. Prostaglandins Other Lipid Mediat. 2019;142:1-8. doi:10.1016/j.prostaglandins.2019.02.007
3.	Molnar V, Matišić V, Kodvanj I, et al. Cytokines and Chemokines Involved in Osteoarthritis Pathogenesis. Int J Mol Sci. 2021;22(17):9208. doi:10.3390/ijms22179208
4.	Glasson SS, Blanchet TJ, Morris EA. The surgical destabilization of the medial meniscus (DMM) model of osteoarthritis in the 129/SvEv mouse. Osteoarthritis Cartilage. 2007;15(9):1061-1069. doi:10.1016/j.joca.2007.03.006
5.	Timkovich AE, Sikes KJ, Andrie KM, et al. Full and Partial Mid-substance ACL Rupture Using Mechanical Tibial Displacement in Male and Female Mice. Ann Biomed Eng. 2023;51(3):579-593. doi:10.1007/s10439-022-03065-1
6.	Foley PL, Liang H, Crichlow AR. Evaluation of a sustained-release formulation of buprenorphine for analgesia in rats. J Am Assoc Lab Anim Sci. 2011;50(2):198-204.
7.	Pezzanite LM, Timkovich AE, Sikes KJ, et al. Erythrocyte removal from bone marrow aspirate concentrate improves efficacy as intra-articular cellular therapy in a rodent osteoarthritis model. Ann Transl Med. 2023;11(9):311-311. doi:10.21037/atm-22-4256
```
