# Supplementary material for: Clonal articular cartilage progenitors outperform mesenchymal stromal cells in mitigating post-traumatic osteoarthritis
Source: Osteoarthr Cartil Open. 2026 Sep 25;8(4):100878. doi: 10.1016/j.ocarto.2026.100878 (PMC13683781; doi:10.1016/j.ocarto.2026.100878)
Supplement: Supplementary file 9 [file mmc2.docx]

**Supplementary Figure 1. Macrophages Stimulated with Pro-inflammatory Cytokines.** Multiplex immunoassay of equine monocyte-derived macrophage cell culture supernatant measuring inflammatory cytokine response after IL-1β, IL-1β + (tumor necrosis factor) TNF, or LPS stimulation. Controls are unstimulated macrophages. Unmeasurable values are reported as zero and signified as an empty shape. **A.** Pro-inflammatory cytokines. **B.** Anti-inflammatory cytokines. **Statistics**: line at mean, error bars mean ± 95% CI, calculated with an ordinary one-way ANOVA & Tukey’s multiple comparisons test with Prism GraphPad version 10. For p-values, means, 95% CI’s, and all statistics, please refer to **Supplementary Tables 4 and 5**. **Acronyms**: IL-1β (interleukin 1 beta), TNF (tumor necrosis factor), interleukin 10 (IL-10), CCL11 (C-C motif chemokine 11), MSCs (mesenchymal stromal cells), ACPs (articular cartilage progenitor cells), LPS (lipopolysaccharide).

**Supplemental Figure 2.** **ANY-maze Open Field Overhead Cage Monitoring.** **A-C.** Longitudinal and summary stats representation of animal behavior and mobility. Data was collected with two baselines prior to injury (averaged), and at weeks 2, 4, and 8 post-injury. The left plots (**i.**) show longitudinal (over time) raw measures. The right plots (**ii.**) show measures at weeks 2, 4 and 8 normalized to baseline (showing differences in scores from the averaged baseline scores of each animals). **A.** Time Mobile (seconds), the amount of time the animal was moving. This includes the time the animal was hidden in the security hut. **B.** Time Immobile Visible (seconds), the amount of time the animals spent not moving not in the security hut. **C.** Average Speed While Moving (meters per second), the calculated average speed while the animal was moving and visible. **D.** A representative image of the ANY-maze apparatus setup and tracking**.** **Statistics**: line at mean, error bars mean ± 95% CI, calculated with a linear mixed model, with multiple comparisons in Prism GraphPad version 10, p values, means, and 95% CIs can be found in **Table 2.** For additional statistics, see **Supplementary Table 6**. **Acronyms**: MSCs (mesenchymal stromal cells), ACPs (articular cartilage progenitor cells), LRS (Lactated ringer’s solution) vehicle control.

**Supplemental Figure 3. DigiGait Footfall Patterns.** Longitudinal and summary stats representation of animal behavior and mobility. Data was collected with two baselines (averaged) prior to injury, and at weeks 2, 4, and 8 post-injury. The left plots (**i.**) show longitudinal (over time) raw measures. The right plots (**ii.**) show measures at weeks 2, 4 and 8 normalized to baseline (showing differences in scores from the averaged baseline scores of each animals). **A.** Stance Width (centimeters), the distance between hind limbs. **B.** Stride Length (centimeters), the distance of the step from one footfall to the next. **C.** Gait Symmetry (ratio), the ratio of forelimb stepping to hind limb stepping frequency. Statistics: line at mean, error bars mean ± 95% CI, calculated with a linear mixed model, with multiple comparisons in Prism GraphPad version 10, p values, means, and 95% CIs can be found in **Table 3.** For additional statistics, see **Supplementary Table 7**. Acronyms: MSCs (mesenchymal stromal cells), ACPs (articular cartilage progenitor cells), LRS (Lactated ringer’s solution) vehicle control.

**Supplemental Figure 4. DigiGait Treadmill Running Schematic**. **A.** DigiGait^TM^ Paw View of Treadmill Running, Mouse running on treadmill orientation from underneath, **B. Baseline treadmill running** representative paw image. (**C-D**) Post-injury treadmill running, with a representative image of **flat-foot running (C)**, and a representative image of **toe-touch running (D)**. (**E-F**) DigiGait^TM^ paw tracking of the right hind limb with the paw area(cm^2^) on the y-axis and the time in seconds on the x-axis. Figures represent **consecutive steps (E)**, the **breaking phase (F)** highlighted by the box with a representative image of the paw on the treadmill while breaking, the **propelling phase (G)** the box highlights the propelling phase of the step, the longest phase of the step, the representative image is of the paw mid-way through the propelling phase, and the **peak stance (H)** where the mouse is neither breaking nor propelling with the paw.

**Supplementary Figure 5. Histopathology Scores of the Medial Compartment of the Right Hind Limb at 8 weeks post DMM. A.** Medial compartment scores: the compiled scoring for the entire medial compartment, sum of domains 1, 2, and 4 as seen in **Supplementary Table 1**. **B.** Medial tibia plateau score: the compiled scores of the joint surface for the medial tibia plateau domains 1 and 2. **C.** Medial femur scores: the compiled scores of the joint surface for the medial femur domains 1 and 2. **D.** Medial synovial and joint capsule score: the compiled scores of the joint capsule and synovium domain 4. **Statistics**, line at mean, error bars mean ± 95% CI, one-way-ANOVA Kruskal-Wallis test—Prism GraphPad v10. **E.** Descriptive statistics table for all joint scoring. **Acronyms**: DMM (destabilization of medial meniscus), MSCs (mesenchymal stromal cells), ACPs (articular cartilage progenitor cells), LRS (Lactated ringer’s solution) vehicle control.

**Supplementary Table 4: Multiplex Statistics Means and 95% CIs**

**Supplementary Table 5: Multiplex All Statistics**

**Supplementary Table 6: ANY-Maze^TM^ Overhead Cage Monitoring All Statistics**

**Supplementary Table 7: DigiGait^TM^ Gait All Statistics**

**Supplementary Table 8: Histology All Statistics**
